# Multiplexed engineering glycosyltransferase genes in CHO cells via targeted integration for producing antibodies with diverse complex-type N-glycans

**DOI:** 10.1038/s41598-021-92320-x

**Published:** 2021-06-21

**Authors:** Ngan T. B. Nguyen, Jianer Lin, Shi Jie Tay, Mariati, Jessna Yeo, Terry Nguyen-Khuong, Yuansheng Yang

**Affiliations:** Bioprocessing Technology Institute, Agency for Science, Technology and Research (A*STAR), Singapore, Singapore

**Keywords:** Antibody therapy, Glycosylation

## Abstract

Therapeutic antibodies are decorated with complex-type N-glycans that significantly affect their biodistribution and bioactivity. The N-glycan structures on antibodies are incompletely processed in wild-type CHO cells due to their limited glycosylation capacity. To improve N-glycan processing, glycosyltransferase genes have been traditionally overexpressed in CHO cells to engineer the cellular N-glycosylation pathway by using random integration, which is often associated with large clonal variations in gene expression levels. In order to minimize the clonal variations, we used recombinase-mediated-cassette-exchange (RMCE) technology to overexpress a panel of 42 human glycosyltransferase genes to screen their impact on antibody N-linked glycosylation. The bottlenecks in the N-glycosylation pathway were identified and then released by overexpressing single or multiple critical genes. Overexpressing B4GalT1 gene alone in the CHO cells produced antibodies with more than 80% galactosylated bi-antennary N-glycans. Combinatorial overexpression of B4GalT1 and ST6Gal1 produced antibodies containing more than 70% sialylated bi-antennary N-glycans. In addition, antibodies with various tri-antennary N-glycans were obtained for the first time by overexpressing MGAT5 alone or in combination with B4GalT1 and ST6Gal1. The various N-glycan structures and the method for producing them in this work provide opportunities to study the glycan structure-and-function and develop novel recombinant antibodies for addressing different therapeutic applications.

## Introduction

The Fc region of IgGs possesses two identical N-glycans which are composed of a core heptasaccharide with three mannoses and two N-acetylglucosamine (GlcNAc). The core structure is further decorated with various sugar like fucose, bisecting GlcNAc, galactose and sialic acid to form a complex-type bi-antennary glycan. N-glycosylation is a critical quality attribute for therapeutic monoclonal antibodies (mAbs), since it affects the drug efficacy, safety, and pharmacokinetic properties^1,2^. The fucosylation of N-glycans is known to negatively impact the antibody dependent cellular cytotoxicity (ADCC) of IgG^3,4^. It has been shown that afucosylated antibodies have 100-fold increased ADCC compared to the fucosylated ones^3^. The galactose moiety of N-glycan is less critical to ADCC than the fucose residue, while its impact on complement dependent cytotoxicity (CDC) is significant^5^. Several studies have demonstrated that increasing galactosylation of antibodies boosted CDC for IgG1 and IgG3 subclasses especially^6,7^. On the contrary, the presence of terminal sialic acid residues on N-glycans neutralized the enhanced CDC activity of the galactosylated mAbs^8^. Sialylation also significantly impaired ADCC of the fucosylated mAbs while it did not adversely change ADCC of the fucose-free IgGs^9–11^. Though removing sialic acid seems to be beneficial for enhancing the Fc effector functions, de-sialylated antibodies displayed poorer pK with faster serum clearance and shorter half-life, potentially dampening drug efficacy^12^. Bas et al. have provided evidence that hyper-sialylated N-glycans significantly increased the IgG serum persistence in the animal model^13^. The terminal sialic acids could also enhance the anti-inflammatory function of antibodies up to 10-fold^14,15^. Thus, sialylated N-glycans on antibodies are also desirable in many therapies like intravenous immunoglobulin (IVIG). It has been recognized that a specific N-glycan profile is favourable over others, depending on the therapeutic applications of antibodies. Being able to engineer a diverse range of N-glycan structures on antibodies will be of great advantage for different therapeutic purposes.

Chinese Hamster Ovary (CHO) cell is the most commonly used host cell for industrial production of therapeutic IgGs due to their capability to perform proper protein folding, assembly of complexes and human-like glycosylation^16,17^. However, wild-type CHO cells generally produce antibodies with incomplete N-glycans that are often highly fucosylated but severely lacking galactose, bisecting GlcNAc and terminal sialic acids^18,19^. To improve complex N-glycan processing, it is necessary to engineer the CHO N-glycosylation pathway and this is often done by overexpression of glycosyltransferase genes in CHO cells. Several studies have shown that overexpression of α-2,6-sialyltransferase 1 (ST6Gal1) gene alone^20,21^ or in combination with galactosyltransferase gene^18,19,22^ in CHO cells could produce antibodies carrying more sialylated N-glycans. However, the achieved sialylation improvement in these studies has been limited. Overexpression of other glycosyltransferase genes that regulate precursor biosynthesis^23,24^, nucleotide sugar transport^25^ and branching extension^26^ alone in CHO cells have been shown to increase sialylated N-glycan structures on erythropoietin (EPO) and IFNγ proteins. Since glycoproteins differ in the positions and numbers of N-glycosylation sites, it is unclear whether the above strategies, which are effective in producing complex N-glycans on recombinant proteins, will work for antibodies as well. In addition, some glycosyltransferase enzymes in the N-glycan biosynthesis pathway have multiple isoforms that could contribute differently to the N-glycan processing in both protein- and site-specific manner^27–29^. Better understanding of the distinct functions of different isoenzymes will provide new opportunities to engineer proteins with more diverse types of N-glycan structures.

Engineering the N-glycosylation pathway by overexpression of glycosyltransferase genes in CHO cells has often been achieved via random integration^18,20,24–26^. The generated stably transfected cell pools exhibit highly heterogeneous gene expression and clonal variation^30,31^, which has limited the success of achieving desirable glycosylation outcomes. Targeted integration approach could overcome such issues by directing transgenes integration into specific loci in genome. As all targeted integration cells have the same genetic background, the obtained stably transfected cells exhibited high levels of phenotypic and transcriptional homogeneity^32^. Targeted integration of transgenes has often been achieved by using recombinase-mediated-cassette-exchange (RMCE). The technique works through the action of recombinases which recognize the specific recombination sites that are pre-determined in the genome and replace the sequence between the two non-compatible sites with a new transgene^33,34^. Among different recombinases, the Flp/FRT system has been extensively applied in CHO cells to express monoclonal antibodies^35^ and recombinant proteins^34,36^ probably due to its high specificity, integration efficiency and low cytotoxity.

In this study we utilized a Flp/FRT-based RMCE system to first overexpress a panel of 42 human glycosyltransferase genes in CHO cells to screen their impact on the N-glycosylation of a model IgG antibody, rituximab. These genes are involved in the different steps of the entire core N-glycosylation pathway and have diverse functions, ranging from sugar precursor synthesis, glycan processing, branching, galactosylation to terminal capping (Table 1). The critical genes that significantly influenced the IgG N-glycosylation were identified and further overexpressed in combination to achieve an array of complex-type N-glycan structures. We also demonstrated that the enzyme expression level was critical in determining glycosylation outcomes and combinatorial engineering of multiple enzymes was required for obtaining highly complex N-glycan structures on mAbs.

**Table 1:** List of human glycosyltransferase genes used in the study.

Group	Name	Accession No	Gene
Nucleotide sugar synthesis	*GALE*	NM_000403	UDP-galactose 4-epimerase
	*GNE*	NM_005476.5	UDP-*N*-acetylglucosamine-2 epimerase (Human)
	*NANS*	NM_018946.3	Sialic acid synthase
	*NANP*	NM_152667.2	*N*-acetylneuraminic acid phosphatase
	*CMAS*	NM_018686.5	Cytidine monophospho-sialic acid synthase
	*CST*	NM_006416.4	CMP-sialic acid transporter
Nucleotide sugar transporter	*UGT*	NM_005660.2	UDP-galactose transporter
	*UGNT*	NM_032826.4	UDP-*N*-acetylglucosamine transporter
	*GFT*	NM_018389.4	GDP-fucose transporter
	*GANC*	NM_198141.2	Neutral α-glucosidase C
	*MANEA*	NM_024641	Endo-α mannosidase
	*MAN1A1*	NM_005907	Mannosyl-oligosaccharide 1,2-α-mannosidase IA
	*MAN1B*	AF_027156	Mannosyl-oligosaccharide 1,2-α-mannosidase IB
Glycan- processing glycosidase	*MAN1C1*	AF_261655	Mannosyl-oligosaccharide1,2-α-mannosidase IC (isoform 1)
	*MAN2A2*	NM_006122.2	α-mannosidase 2A member 2
	*MAN2B1*	NM_000528.3	α-mannosidase, class 2B, member 1
	*MAN2B2*	NM_015274.2	α-mannosidase, class 2B, member 2
	*MAN2C1*	NM_006715.3	α-mannosidase, class 2C, member 1
	*MGAT1*	NM_001114618.1	α-1,3-mannosyl-glycoprotein 2-β-*N*-acetylglucosaminyltransferase
	*MGAT2*	BC_006390	α-1,6-mannosyl-glycoprotein 2-β-*N*-acetylglucosaminyltransferase
	*MGAT3*	NM_002409.4	β-1,4-mannosyl-glycoprotein 4-β-*N*-acetylglucosaminyltransferase
N-Glycan chain extension	*MGAT4A*	NM_012214.2	α-1,3-mannosyl-glycoprotein 4-β-*N*-acetylglucosaminyltransferase A
	*MGAT4B*	AB_000624	α-1,3-mannosyl-glycoprotein 4-β-*N*-acetylglucosaminyltransferase B
	*MGAT4C*	BC_064141	α-1,3-mannosyl-glycoprotein 4-β-*N*-acetylglucosaminyltransferase C
	*MGAT5*	NM_002410.4	α-1,6-mannosyl-glycoprotein 6-β-*N*-acetylglucosaminyltransferase
	*MGAT5B*	NM_144677.2	α-1,6-mannosyl-glycoprotein 6-β-*N*-acetylglucosaminyltransferase B
	*B4GALT1*	NM_001497.3	β-1,4-Galactosyltransferase 1
	*B4GALT2*	NM_030587.2	β-1,4-Galactosyltransferase 2
	*B4GALT3*	NM_001199873.1	β-1,4-Galactosyltransferase 3
Galactosylation	*B4GALT4*	NM_212543.1	β-1,4-Galactosyltransferase 4
	*B4GALT5*	NM_004776.3	β-1,4-Galactosyltransferase 5
	*B4GALT6*	NM_004775.3	β-1,4-Galactosyltransferase 6
	*B4GALT7*	NM_007255.2	β-1,4-Galactosyltransferase 7
	*ST3GAL1*	NM_174963.3	β-Galactoside -α-2,3-sialyltransferase 1
	*ST3GAL2*	NM_006927.3	β-Galactoside -α-2,3-sialyltransferase 2
	*ST3GLA3*	NM_174963.3	β-Galactoside -α-2,3-sialyltransferase 3
Sialylation	*ST3GAL4*	NM_001254757.1	β-Galactoside -α-2,3-sialyltransferase 4
	*ST3GAL5*	NM_003896.3	β-Galactoside -α-2,3-sialyltransferase 5
	*ST3GAL6*	NM_006100.3	β-Galactoside -α-2,3-sialyltransferase 6
	*ST6GAL1*	BC_040009	β-Galactoside -α-2,6-sialyltransferase 1
	*ST6GAL2*	NM_032528.2	β-Galactoside -α-2,6-sialyltransferase 2
Fucosylation	*FUT8*	NM_178155.2	α-1,6-Fucosyltransferase

## Results

### Design of RMCE to screen for functional roles of human glycosyltransferase genes in N-glycosylation of antibodies in CHO cells

**Figure 1:**
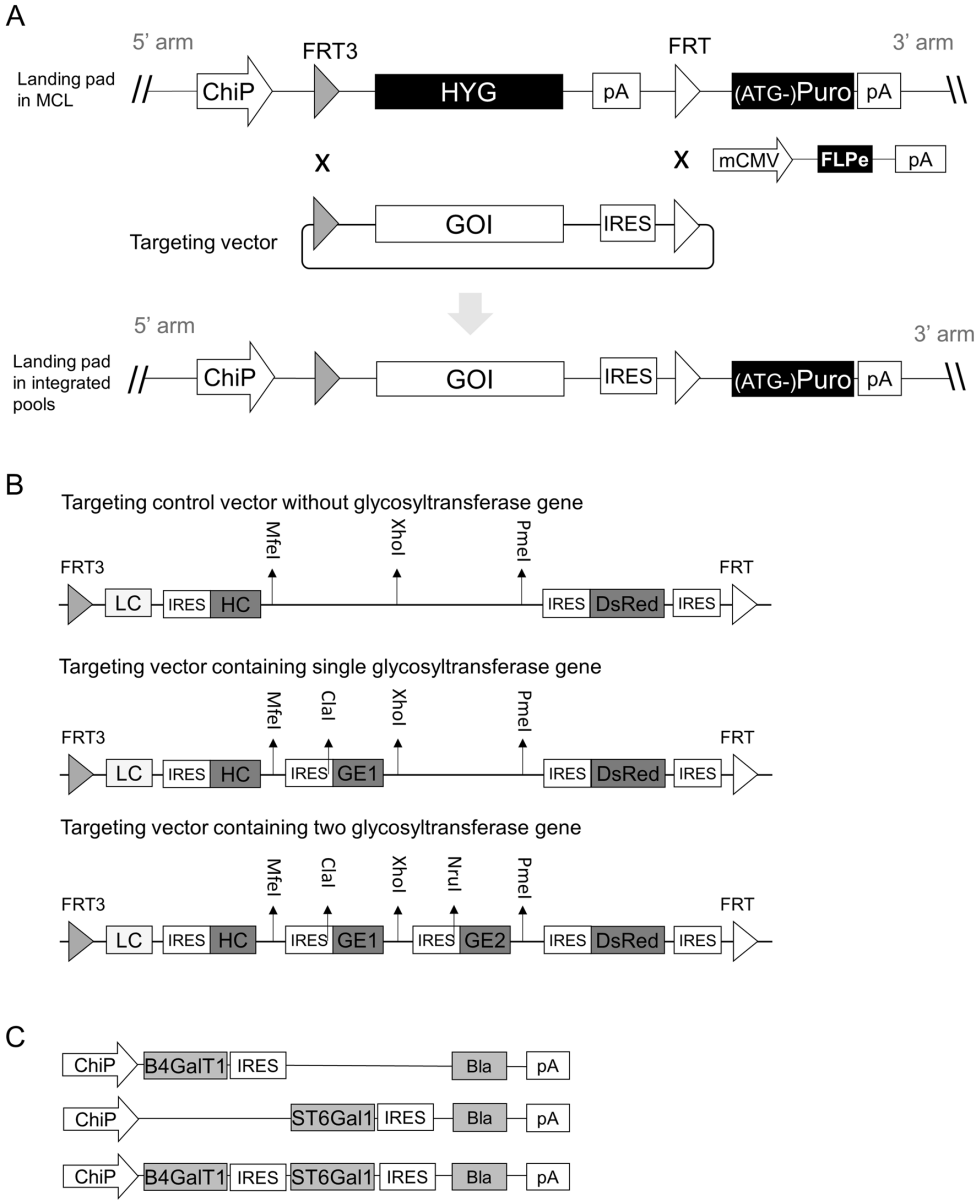
Overview of RMCE and plasmid vectors for the generation of stably transfected CHO cell pools that co-express IgG and human glycosyltransferase genes. (**A**) Schematic representation of RMCE. (**B**) Schematic representation of targeting plasmid vectors carrying IgG rituximab LC and HC genes together with one or two human glycosyltransferase genes and DsRed gene. (**C**) Schematic representation of plasmid vectors expressing B4GalT1 and ST6Gal1 individually or in combination for random integration. RMCE, recombinase-mediated-cassette-exchange; MCL, master cell line; GOI, gene of interest; ChiP, a chimeric promoter consisting of murine cytomegalovirus (CMV) enhancer, human CMV core promoter and human CMV intron A; mCMV, murine CMV enhancer and promoter; FRT and FRT3, wild-type and mutated flippase recognition targets; Flpe, enhanced recombinase flippase; IRES, wild-type encephalomyocarditis virus (EMCV) internal ribosome entry site (IRES); pA, simian virus 40 polyadenylation signal; LC, light chain cDNA; HC, heavy chain cDNA; G.E.1, glycosyltransferase gene 1; G.E.2, glycosyltransferase gene 2; DsRed, cDNA encoding red fluorescent protein; (ATG-)Puro, start-codon ATG-deleted puromycin resistence gene; Bla, blasticidin-S resistance gene; B4GalT1, β-1,4-Galactosyltransferase 1 gene; ST6Gal1, β-Galactoside -α-2,6-sialyltransferase 1 gene.

In order to study the impact of overexpressing human glycosyltransferase genes on IgG glycosylation, we co-expressed the individual glycosylation gene together with the antibody gene in a CHO K1 master cell line (MCL) via RMCE-based targeted integration (Fig. 1A). The MCL was confirmed to have one single integrant of a landing pad vector by Southern blot and targeted locus amplification (TLA) analysis (data not shown). The landing pad vector contained a hygromycin resistant gene (HYG) flanked by a wild-type flippase recognition target (FRT) and its mutant FRT3. An impaired puromycin resistant gene lacking a start codon ((ATG-)Puro) was placed downstream of FRT. The transcription of HYG gene was driven by a chimeric promoter (ChiP) and terminated by a SV40 polyadenylation signal (pA) upstream of FRT. As a result, the impaired (ATG-)Puro was not expressed before activation by RMCE. Each targeting vector was designed to be promoter-less and carry the rituximab light chain (LC) and heavy chain (HC) genes, together with one or two specific human glycosyltransferase genes and the DsRed gene linked through multiple wild-type encephalomyocarditis virus (EMCV) internal ribosome entry site (IRES) (Fig. 1B). An additional wild-type EMCV IRES was placed downstream of DsRed for activating the expression of (ATG-)Puro upon replacing the HYG cassette in the MCL through RMCE. The targeting vector carrying only rituximab and DsRed genes served as the control. Rituximab is a therapeutic IgG1 mAb for treating rheumatoid arthritis and B-cell non-Hodgkin's lymphoma^37^.

RMCE was carried out by co-transfection of a specific targeting vector and the vector expressing an enhanced recombinase flippase (FLPe) followed by puromycin selection to obtain stably transfected pools. As the targeting vectors did not have promoter and polyadenylation signal, they could not express the carried genes if they randomly integrated into the chromosomes. The combination of promoter-trap and ATG-trap ensured that only cells with correct RMCE expressing all genes carried on the targeting vectors can survive the puromycin selection. As all targeted cells in a stably transfected pool share the same integration site, variation caused by position effect and gene copy number could be potentially minimized^32,38^, which enabled studying gene functions in a pool of transfected cells without the need of isolating single cell clones. In contrast to using multiple promoters for co-expression of multiple genes, using IRES has advantage of allowing independent expression of multiple genes in one transcript without causing interference to each others^39^. Inclusion of the DsRed gene in each targeting vector permitted easy analysis of the homogeneity of gene expression in a population of targeted cells by FACS.

### Identification of critical human glycosyltransferase genes which overexpression affect antibody N-glycosylation in CHO cells

**Figure 2:**
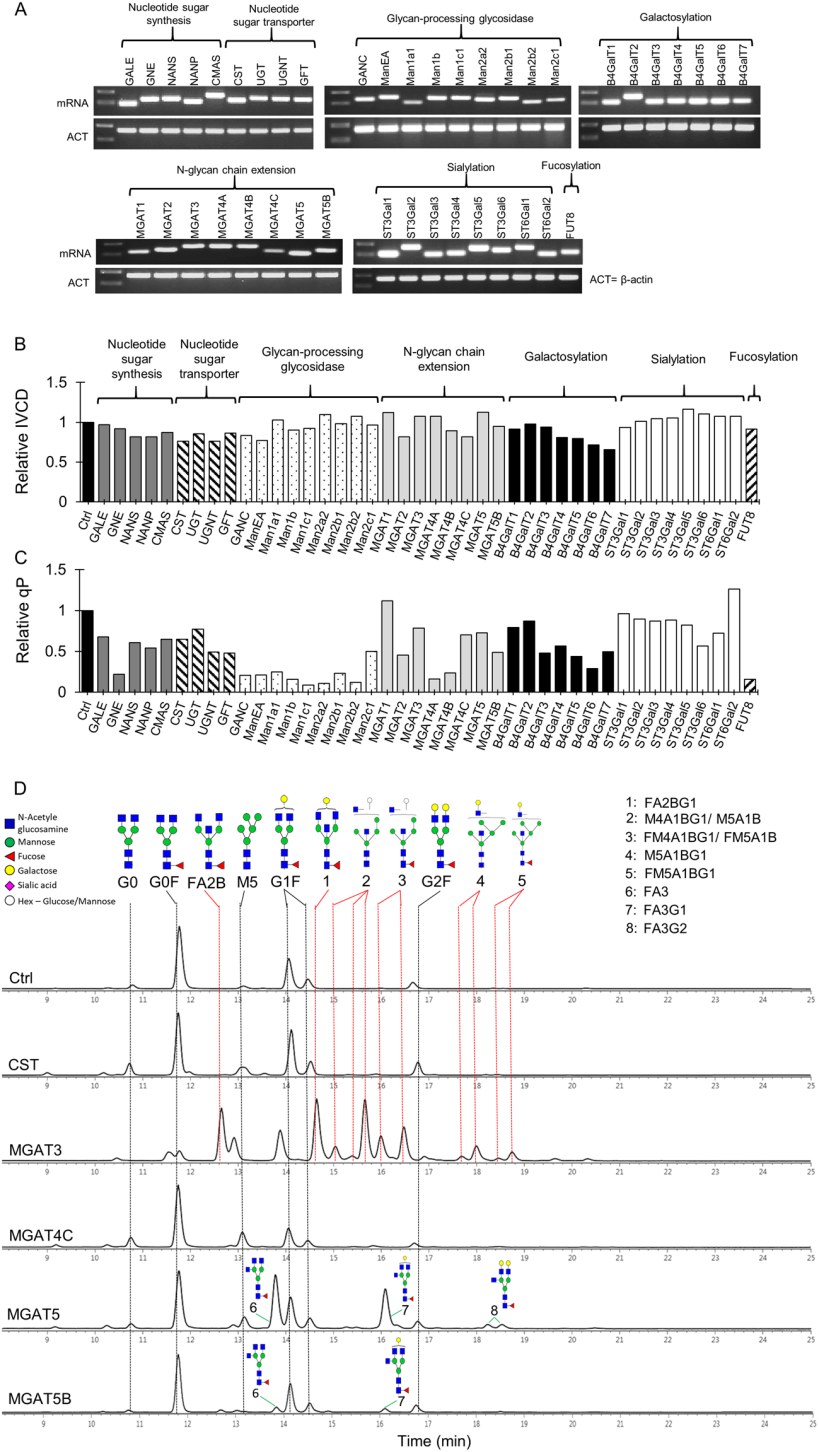

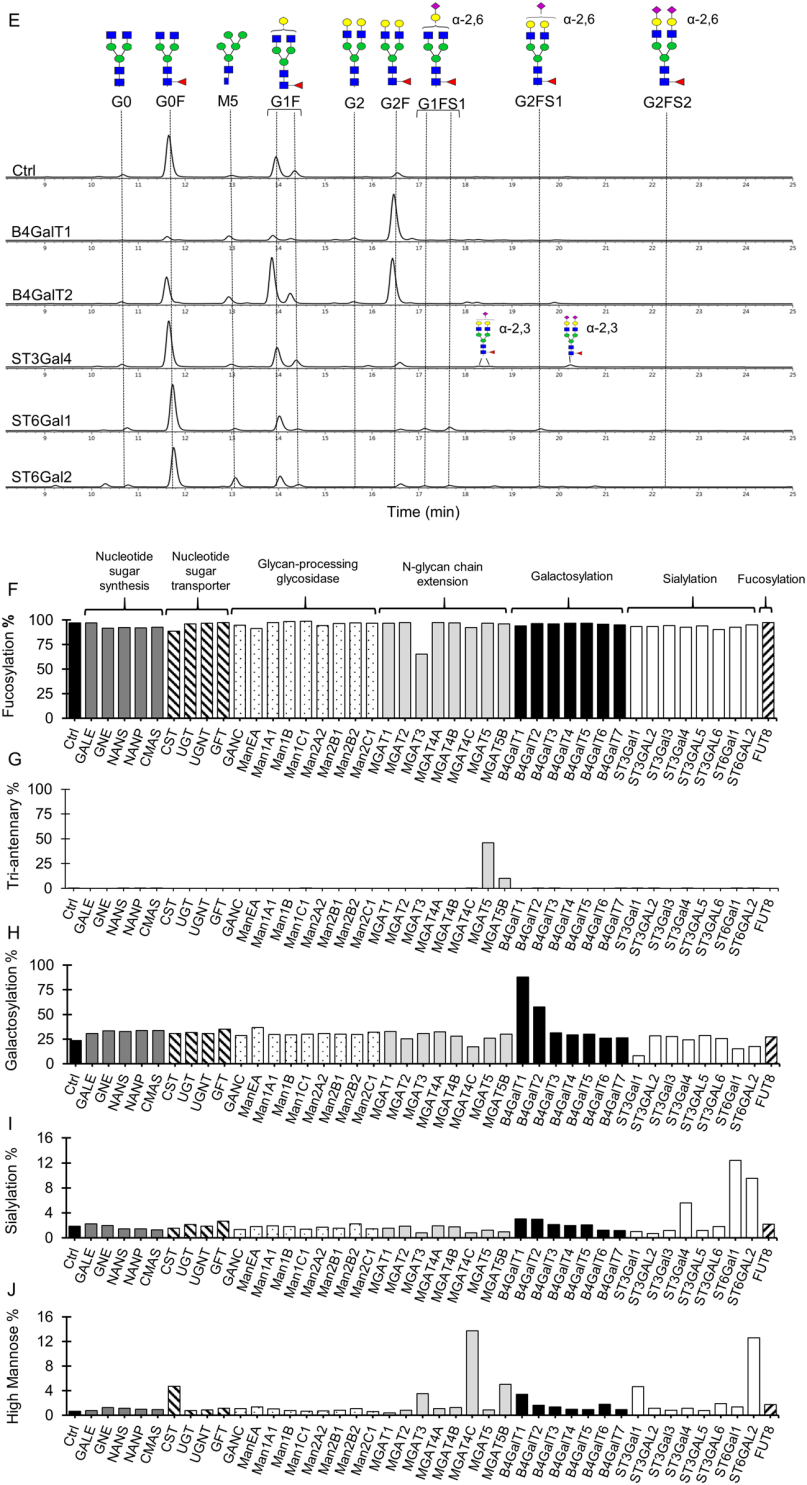
Impact of overexpressing individual human glycosyltransferases on growth, productivity and N-linked glycosylation of antibodies produced in stably transfected CHO cell pools. All stable pools were generated via RMCE. The control (Ctrl) pool expressed only IgG1 rituximab LC, HC and DsRed genes. Each of the other 42 stable pools co-expressed IgG1 rituximab genes, a specific human glycosyltransferase gene and DsRed. (**A**) RT-PCR analysis of human glycosyltransferase transcripts in different stable pools. β-actin (ACT) was used as an internal control. (**B**–**C**) Relative change in the integrated viable cell density (IVCD) and specific productivity (qP) in each stably transfected pool to the control. (**D**–**E**) Aligned HILIC chromatograms of N-linked glycans on antibodies in the control and representative stable pools which exhibited changes in the N-glycan profiles. Symbolic representation of the N-glycan structures was depicted for dominant peaks. Red dotted line indicated the N-glycan structures that were only found in MGAT3 sample. (**F**–**J**) Relative distribution of fucosylation, galactosylation, tri-antennary, sialylation and high-mannose on antibodies produced in different stable pools.

In total, a panel of 42 human glycosyltransferases in the core N-glycosylation pathway were selected in this study (Table 1). They were categorized into eight groups based on their known functions: nucleotide sugar synthesis, nucleotide sugar transport, glycan-processing glycosidases, N-glycan chain extension, galactosylation, sialylation and fucosylation. The impact of each gene on cell growth, antibody productivity and N-glycan profiles was studied by generating 42 stably transfected pools expressing individual genes through RMCE (Fig. 1). The correct integration of each targeting vectors into the landing pad was verified by the 5′ and 3′ junction PCRs (Supplementary Fig. S1) using two pairs of primers specifically binding to the region in the landing pad vector and the targeting vector, respectively (Table S1). Analysing mRNA by RT-PCR confirmed successful overexpression of all human glycosyltransferase genes in the stably transfected pools (Fig. 2A). Moreover, FACS analysis of the stable pools indicated that the targeted cells had homogenous DsRed expression levels (Supplementary Fig. S2), which certified the use of targeted pools for studying gene functions and avoided the need of isolating clones. Each stable pool was characterized for growth, antibody expression level and N-glycosylation in 7-day fed-batch cultures. Adding feed medium and harvesting samples at exponential growth phase excluded the possible effect of nutrients depletion on antibody expression and glycosylation. Overexpressing each glycosylation gene caused little change in cell growth as indicated by the integrated viable cell density (IVCD) compared to the control culture (Fig. 2B). In contrast, significant decreases in specific antibody productivity (qP) were observed in stable pools overexpressing many glycosylation genes (Fig. 2C). All glycan-processing glycosidases decreased qP ranging from 0.1 to 0.5-fold of the control culture. Majority of the genes involved in other parts of the glycosylation pathway exhibited less effect on qP except for a few particular genes, such as GNE, MGAT4A, MGAT4B and FUT8 which overexpression resulted in decreased qP to about 0.2-fold of the control culture. Since folding and assembly of antibody LC and HC occur in the Endoplasmic reticulum (ER), overexpression of ER-resident enzymes like mannosidases may negatively affect folding of antibodies, and possibly the secretion rate. Further studies are needed to have a clearer understanding of how glycan-processing glycosidases and other genes affect antibody secretion.

The N-linked glycan profiles of Rituximab produced in stable pools overexpressing various human glycosyltransferase genes were analyzed by hydrophilic interaction liquid chromatography (HILIC). The different N-glycosylation components were further quantified as percentage of fucosylation, galactosylation, tri-antennary branching, high-mannose and sialylation. Among the 42 tested genes, only 10 resulted in dramatic shifts in antibody N-glycosylation. The chromatograms of N-glycans for these ten genes together with the control were presented in Fig. 2D,E, while genes with no or little effect on N-glycan profiles were not shown. Consistent with previous studies^18,19^, we observed that the control culture produced antibodies with N-glycans that were mostly G0F, a fucosylated bi-antennary species lacking both galactose and sialic acid (Fig. 2D). The targeted integration pool overexpressing MGAT3 produced IgGs carrying many bisecting glycans such as FA2B and FA2BG1, fucosylated bisecting species without and with galactose, respectively (Fig. 2D). The antibody fucosylation in MGAT3-expressing cells also reduced to 65% as compared to 98% in the control antibody (Fig. 2F). Similarly, overexpressing B4GalT1 produced antibodies containing mainly G2F, bi-galactosylated glycans and overexpressing ST6Gal1 slightly increased G2FS1 and G2FS2, fucosylated bi-antennary species containing one and two α-2,6-sialic acids, respectively (Fig. 2E). Interestingly, we also observed 6 other genes, which impacts on antibody N-glycosylation have not been emphasized in previous studies, significantly changed the N-glycan profiles of antibodies. B4GalT1 has six galactosyltransferase isoenzymes. Only overexpression of B4GalT2 led to significant conversion of G0F species to G1F and G2F, a mono- and bi-galacosylated species (Fig. 2E), although the galactosylation increment was lower compared to that achieved by overexpressing B4GalT1 (Fig. 2H). ST6Gal2 is the only isoenzymes of ST6Gal1, which overexpression also resulted in a 9% increase in the α-2,6-sialic acid content (Fig. 2I). Six isoenzymes of α-2,3-sialyltransferase, that are responsible for incorporating α-2,3-sialic acids into the glycans, were tested. It has been reported that all these isoenzymes are naturally expressed in CHO cells^29^ although sialic acids on antibodies produced in the wild-type CHO cells are minimal^18^. Overexpressing human ST3Gal4 gene resulted in slightly enhanced α-2,3-sialic acids while the other five human sialyltransferase isoenzymes had no effect on antibody N-glycosylation (Fig. 2I). MGAT4 and MGAT5 have been reported to enhance branching of N-glycans on EPO^40^. Interestingly, among three MGAT4 and two MGAT5 isoenzymes, overexpression of MGAT4C enhanced high-mannose while MGAT5 and MGAT5B resulted in production of antibodies containing many tri-antennary glycans (Fig. 2D,G). This is interesting as it is well known that antibody glycans are bi-antennary. Most genes acting in the earlier steps of the N-glycosylation biosynthetic pathway like nucleotide sugar synthesis and transporter showed little impact on the IgG fucosylation, branching, galactosylation and sialylation (Fig. 2F,G,H,I). High-mannose structures were slightly enhanced in a number of cultures overexpressing CST, MGAT4C, MGAT5B, ST3Gal1 and ST6Gal2, though the magnitude was small, ranging from 4 to 10% (Fig. 2J). The increases in high mannoses could be due to different mechanisms. For instance, it is known that ST3Gal1 plays a key role in adding α2,3-linked sialic acid to substrates in O-glycans^41,42^. A previous study observed that inhibition of O-glycosylation pathway increased N-glycan levels, suggesting that the O-glycosylation and N-glycosylation pathways could be interacted and influenced with each another^43^. We speculate that overexpression of ST3Gal1 in CHO cells might enhance the O-glycosylation pathway, which in turn negatively affect the N-glycosylation, thus resulting in increased proportion of high mannose and decreased proportion of galactose. Regarding the increased high mannose by overexpressing MGAT4C and MGAT5B, one possible explanation is that the branching reaction is competing with the later steps of N-glycan extension reactions. The reason for the increased high mannose by overexpression of other enzymes is hardly understood. We speculate that overexpression of glycosylation enzymes at high abundance may result in their relocation in the ER and Golgi, thus resulting in unexpected functions. Further studies are needed to elucidate the roles of these genes in high-mannose glycan formation.

### Combinatorial engineering of complex-type bi-antennary N-glycans on antibodies

**Figure 3:**
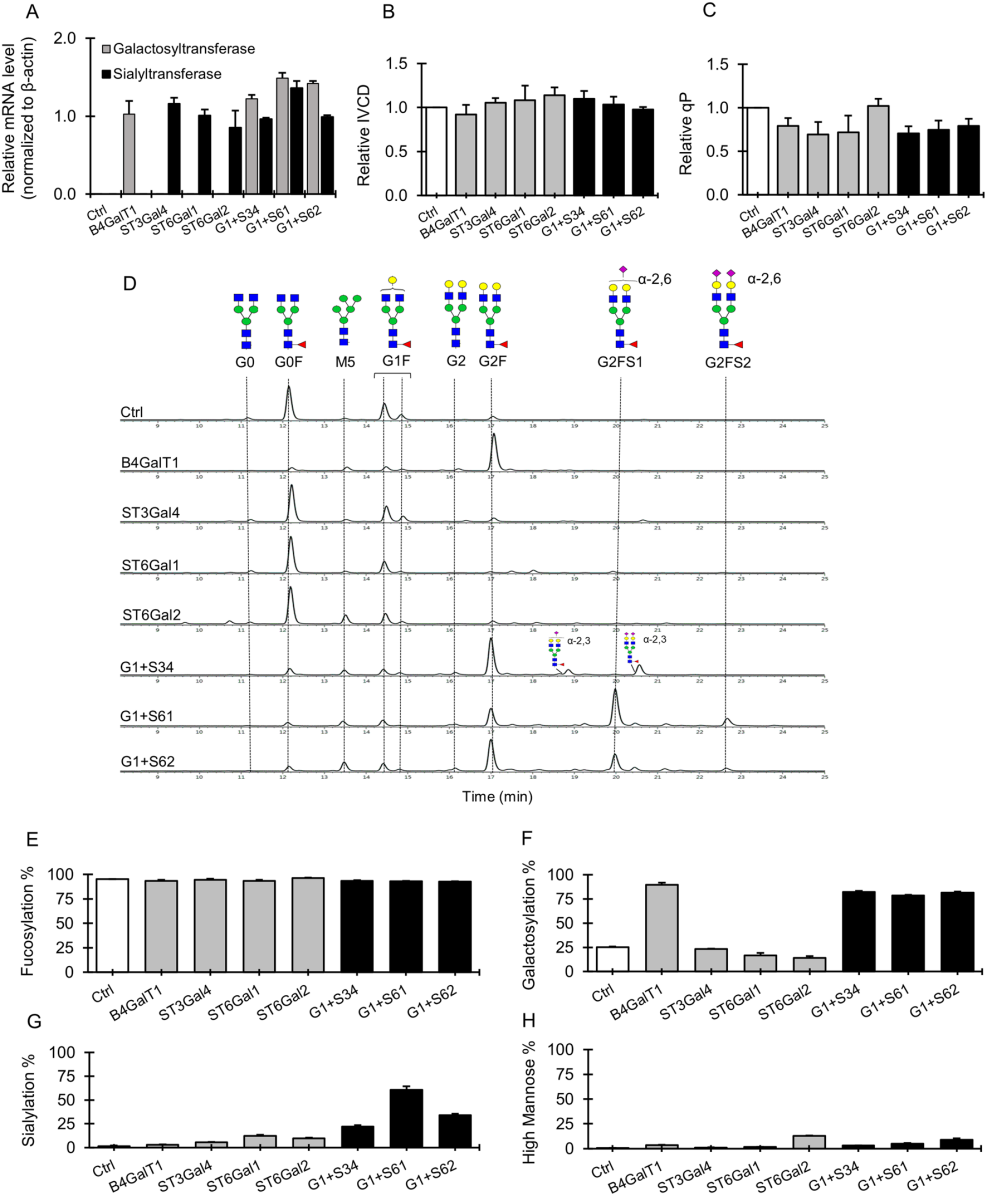
Impact of co-expressing B4GalT1 and sialyltransferase isoenyzmes on the growth, productivity and glycosylation of antibodies in stably transfected CHO cell pools. All stable pools were generated through RMCE. The control pools expressed IgG rituximab LC, HC and DsRed genes. Other stable pools expressed IgG rituximab LC and HC genes, B4GalT1 and ST6Gal1 either individually or in combination, and DsRed. G1 + S34, G1 + S61, G1 + S62 are combinatorial stable pools co-expressing B4GalT1 and either of ST3Gal4, ST6Gal1 and ST6Gal2 gene, respectively. All stable pools were characterized in 7-day fed-batch cultures. (**A**) Relative galactosyltransferase/ sialyltransferase transcript levels in different stable pools to the internal control, β-actin (ACT) as determined by quantitative real time-PCR (qRT-PCR). (**B**–**C**) Relative change in the integrated viable cell density (IVCD) and specific productivity (qP) of each stably transfected pool to the control. (**D**) Aligned HILIC chromatograms of N-linked glycans on antibodies in different stable pools. Chromatograms shown are one of biological replicates with similar results. (**E**–**H**) Relative distribution of fucosylation, galactosylation, sialylation and high-mannose on antibodies produced different stable pools. Each point represents the average and standard deviation of measurements from two independent stably transfected pools.

Overexpression of B4GalT1 gene alone in the wild-type CHO cells converted majority of G0F to G2F glycans on IgGs (Fig. 2E). However little to no sialylated species were observed in the B4GalT1 overexpressing pools. To obtain highly sialylated bi-antennary glycans, we co-expressed each of the three isoenzymes ST3Gal4, ST6Gal1 and ST6Gal2, which were identified to slightly enhance mAb sialylation when being overexpressed individually in CHO cells (Fig. 2E,I), with B4GalT1 in the MCL using targeting vectors and RMCE as described in Fig. 1A,B. Across all the combinatorial pools, the transcript levels of the two glycosyltransferase genes were relatively comparable (Fig. 3A), indicating no transcriptional interference between them. Compared to the single B4GalT1 overexpressing pools, combinatorial overexpression of B4GalT1 with either ST3Gal4 (G1 + S34), ST6Gal1 (G1 + S61) or ST6Gal2 (G1 + S62) did not generate negative impacts on cellular growth IVCD (Fig. 3B) and specific productivity qP (Fig. 3C). mAbs produced in the single B4GalT1 overexpressing pools contained predominantly G2F glycans, while mAbs produced in all the three ST3Gal4, ST6Gal1 and ST6Gal2 single gene overexpressing pools contained mostly G0F and small proportion of G1F glycans (Fig. 3D). Upon co-expressing the B4GalT1 gene and one sialyltransferase gene, we observed a significant increase in the sialylated complex N-glycans, G2FS1 and G2FS2, the mono- and di-sialylated species in the combinatorial pools (Fig. 3D). The overall galactosylation distribution in the combinatorial pools was nearly 80%, similar to that in the single B4GalT1 overexpressing pools (Fig. 3F). The G1 + S61 pools produced the highest sialylation increment up to 60% (Fig. 3G), with majority of N-glycans being G2FS1, followed by a small proportion of G2FS2 species. In contrast, co-expressing the B4GalT1 gene and any one of the three sialyltransferase genes had no significant impact on the levels of fucose and high mannose compared to the control (Fig. 3E,H).

**Figure 4:**
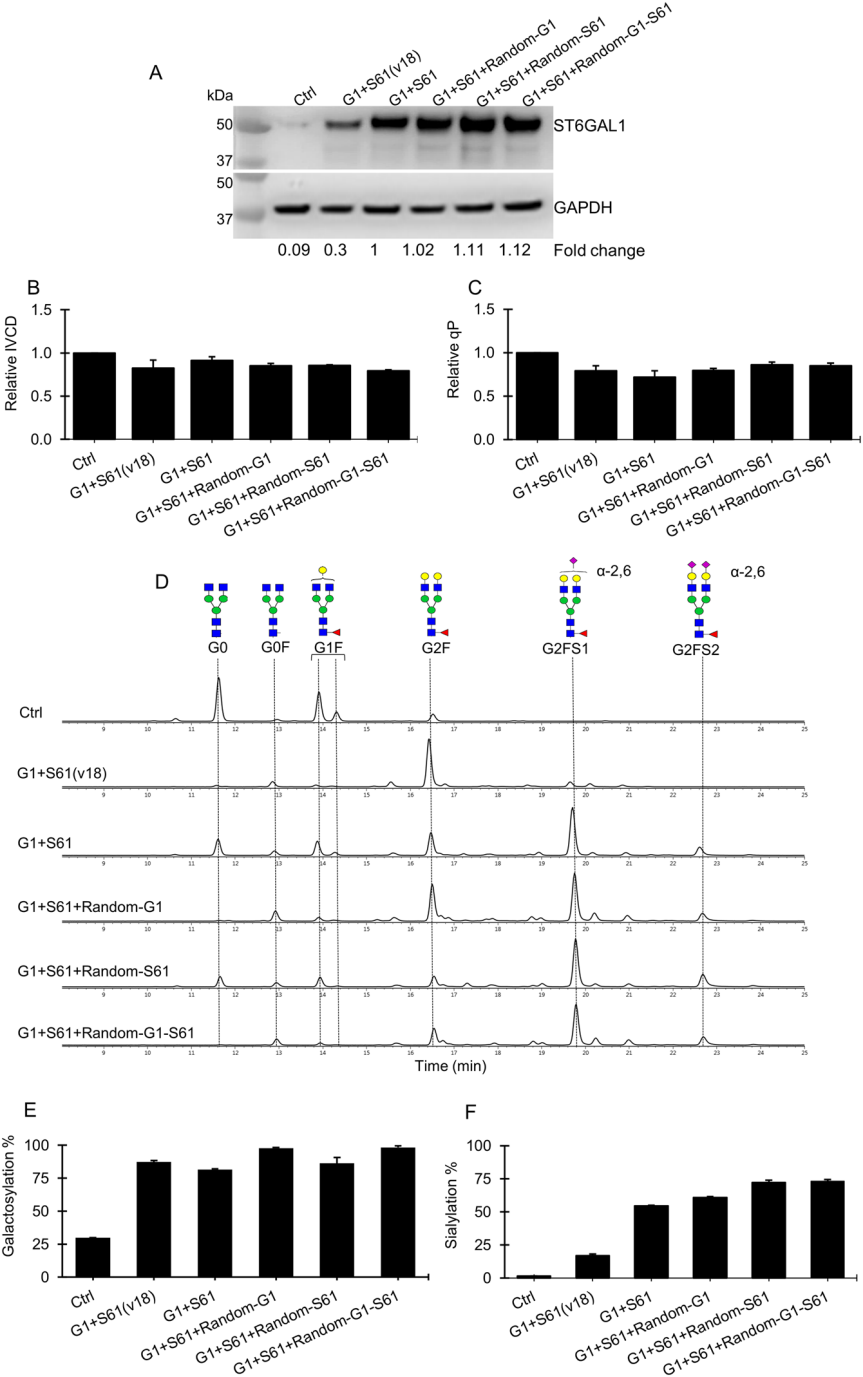
The relationship between the expression level of B4GalT1 and ST6Gal1 and the galactose and sialic acid contents on the antibodies produced in the stably transfected CHO cell pools. The targeted pools of control (Ctrl), G1 + S61 and G1 + S61(v18) were generated using different targeting vectors through RMCE. The control targeting vector expressed IgG rituximab LC, HC and DsRed genes. The two targeted vectors for generating G1 + S61 and G1 + S61(v18) pools were the same, which carried IgG rituximab LC and HC genes, DsRed, B4GalT1 and ST6Gal1, except that the ST6Gal1 in the former vector was controlled by a wild-type IRES while in the latter vector was controlled by a mutant IRESv18 with reduced translation efficiency. The G1 + S61 targeted pools were further transfected with three plasmid vectors expressing B4GalT1 and ST6Gal1 individually or in combination, respectively, followed by selection with blasticidin to generate three sets of stably transfected pools, G1 + S61 + Random-G1, G1 + S61 + Random-S61 and G1 + S61 + Random-G1-S61, for further enhancing B4GalT1 and/or ST6Gal1 expression levels. All stable pools were characterized for growth, productivity and antibody glycosylation in 7-day fed-batch cultures. (**A**) Western Blot analysis of ST6Gal1 protein levels in different stable pools. The housekeeping protein GAPDH was used as a loading control. (**B**–**C**) Relative change in the integrated viable cell density (IVCD) and specific productivity (qP) to the control pool. (**D**) Aligned HILIC chromatograms of N-glycans on antibodies produced in different stable pools. Chromatograms shown are one of biological replicates with similar results. (**E**–**F**) Relative distribution of galactosylation and sialylation level of N-glycans on antibodies produced in different stable pools. Two stably transfected CHO cell pools were generated for each vector. Each point represents the average and standard deviation of measurements from two independent stably transfected pools.

There was still a sizeable proportion of the G2F glycans on antibodies produced in the combinatorial G1 + S61 pools, suggesting that the expression level of sialytransferase was probably insufficient to add more sialic acids to G2F. In order to obtain further increased sialylation, we further overexpressed B4GalT1 and/or ST6Gal1 in the G1 + S61 targeted pools through random integration of three vectors expressing B4GalT1 and ST6Gal1 genes either individually or in combination, respectively (Fig. 1C). This resulted in increased B4GalT1 and/or ST6Gal1 transcripts as shown via RT-PCR (Supplementary Fig. S3A) and elevated overall ST6Gal protein levels (Fig. 4A) in the G1 + S61 + Random-G1, G1 + S61 + Random-S61 and G1 + S61 + Random-G1-S61 stable pools. Compared to the G1 + S61 targeted pools, the newly generated stable pools did not exhibited decrease in the cellular growth IVCD (Fig. 4B) and specific antibody productivity qP (Fig. 4C). The G1 + S61 + Random-G1 pools with additional overexpression of B4GalT1 showed a moderate increase in galactosylation from 80 to 98% and a small increment in sialylation from 60 to 62% (Fig. 4D,E,F). Stacking up ST6Gal1 expression alone in the G1 + S61 + Random-S61 pools increased the sialic acid content to nearly 75% despite no additional enhancement in the total galactose level compared to the G1 + S61 targeted pools. The increased expression levels of both B4GalT1 and ST6Gal1 in the G1 + S61 + Random-G1-S61 pools elevated both galacosylation and sialylation levels. However, the total sialylation in the G1 + S61 + Random-G1-S61 pools was similar to that in the G1 + S61 + Random-S61 pools in spite of the increased galactosylation. These findings suggested that sialylation had become the bottleneck for maximizing sialic acids in G1 + S61 targeted pools. To verify this hypothesis, we generated G1 + S61(v18) targeted pools using a targeting vector which was same as the one for generating G1 + S61 targeted pools except that the ST6Gal1 gene was controlled by a mutant IRESv18 with redueced translation efficiency^39^. Compared to the G1 + S61 targeted pools, the ST6Gal1 expression in the G1 + S61(v18) pools was reduced by 70% (Fig. 4A) while the B4GalT1 gene expression remained similar (Supplementary Fig. S3A). With decreased ST6Gal1 protein level, we observed a corresponded decrease in the total sialylation from 60 to 17% in the G1 + S61(v18) pools while the galactose content remained similar compared to the G1 + S61 targeted pools (Fig. 4E,F). This further supported our hypothesis that the expression level of ST6Gal1 gene was the limiting factor in the G1 + S61 pools for obtaining further enhanced sialic acid content on antibodies.

### Combinatorial engineering of complex-type tri-antennary N-glycan on antibodies

**Figure 5:**
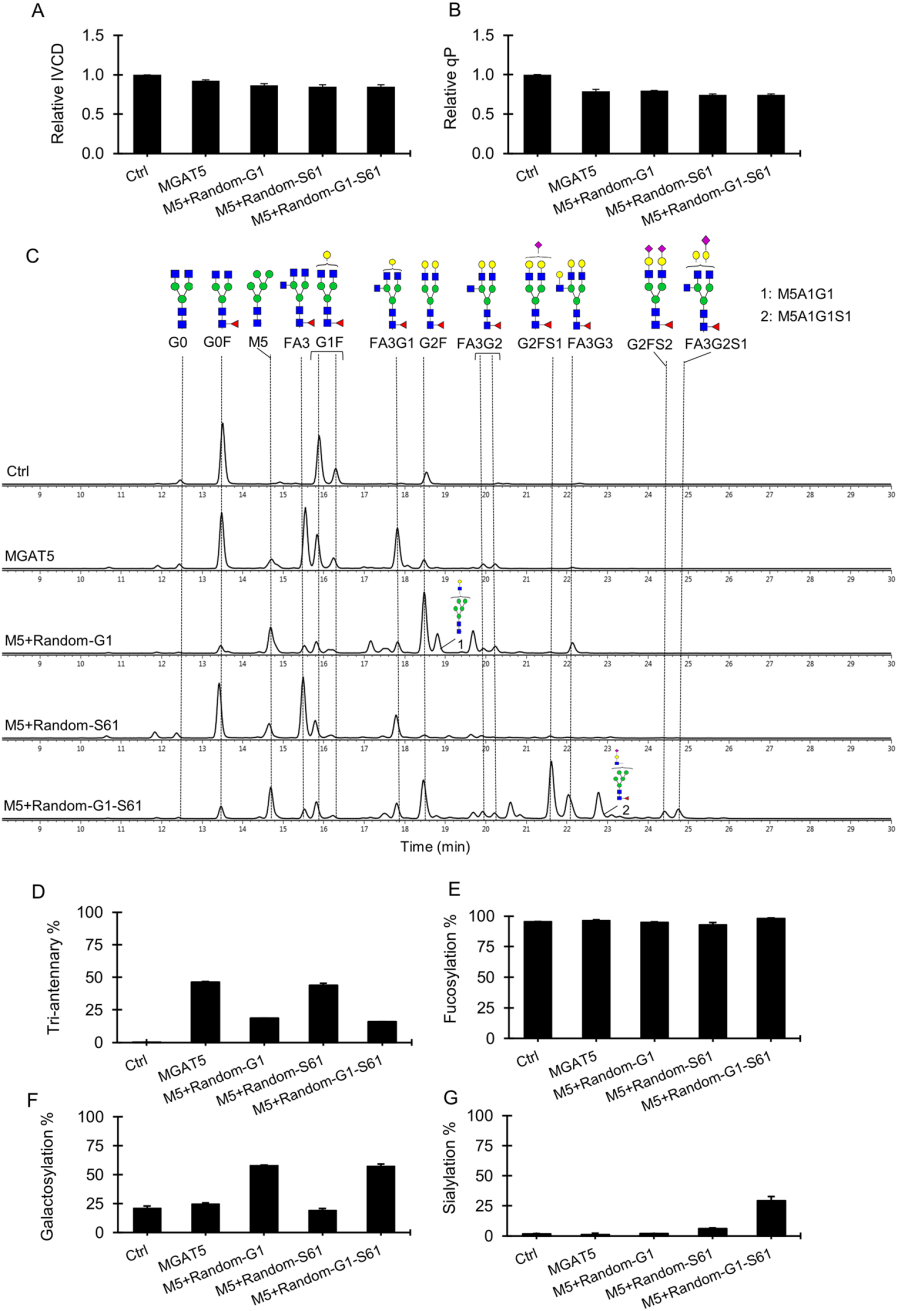
Combinatorial engineering of tri-antennary N-glycans on antibodies for enhanced galactosylation and sialylation. The control (Ctrl) and MGAT5 targeted pools were generated through RMCE. The control targeted pools expressed IgG rituximab LC, HC and DsRed genes. The MGAT5 targeted pools expressed IgG rituximab LC and HC genes, MGAT5 and DsRed. The targeted pools overexpressing MGAT5 were further transfected with three plasmid vectors carrying the B4GalT1 and ST6Gal1 gene individually or in combination, followed by blasticidin selection to generate three sets of stable pools, M5 + Random-G1, M5 + Random-S61 and M5 + Random-G1-S61. All stable pools were characterized for growth, productivity and antibody glycosylation in 7-day fed-batch cultures. (**A**–**B**) Relative change in the integrated viable cell density (IVCD) and specific productivity (qP) to the control pool. (**C**) Aligned HILIC chromatograms of N-glycan profile on antibodies produced in different stable pools. Chromatograms shown are one of biological replicates with similar results. (**D**–**G**) Relative distribution of the tri-antennary, fucosylation, galactosylation, and sialylation levels of N-glycans on antibodies produced in different stable pools. Each point represents the average and standard deviation of measurements from two independent stably transfected pools.

Inspired by the novel finding of tri-antennary N-glycans on mAbs produced by overexpressing MGAT5, we explored the possibility of engineering tri-antennary N-glycans with high galactosylation and sialylation by introducing B4Gal1 and ST6Gal1 genes into the MGAT5 over-expressing pools. To avoid incomplete integration through RMCE due to the large size of the targeting vectors (unpublished data), we utilized the same three vectors expressing B4GalT1 and ST6Gal1 individually or in combination for random integration (Fig. 1C). RT-PCR analysis confirmed successful expression of the newly introduced B4GalT1 and ST6Gal1 transcripts (Supplementary Fig. S3B). Compared to the MGAT5 targeted integration pools, additional expression of B4GalT1 and/or ST6Gal1 in the combinatorial pools resulted in a 10% reduction in IVCD (Fig. 5A) while the specific productivity qP of remained unchanged (Fig. 5B). Antibodies produced in the control pools contained mainly bi-antennary N-glycans, of which more than half were G0F followed by lesser abundance of G1F and G2F species (Fig. 5C). Besides having a similar trended distribution of G0F, G1F and G2F as seen on the control antibodies, antibodies produced in the MGAT5 overexpressing pools also carried a high proportion of tri-antennary N-glycans that were largely agalactosylated (FA3), followed by moderate level of tri-antennary N-glycans containing one galactose (FA3G1) and a small proportion of tri-antennary N-glycans containing two (F3AG2) and three galactoses (FA3G3) (Fig. 5C). The relative distribution of total tri-antennary N-glycans in the MGAT5 pools reached about 50% (Fig. 5D). Further overexpression of B4Gal1 gene in the MGAT5 pools converted most G0F and G1F into G2F glycans. Interestingly, significant decrease in FA3 level was observed but without accompanying the corresponded increase in the galactose content in tri-antennary N-glycans (Fig. 5C). Only slight glycosylation shifts toward tri-antennary glycans that were bi-galactosylated (FA3G2) and tri-galactosylated (FA3G3) were observed in the M5 + Random-G1 pools. As a result, the total tri-antennary N-glycans decreased by half as compared to the MGAT5 pools (Fig. 5D). Compared to the MGAT5 N-glycan distribution, there were little changes to the glycan profile in the M5 + Random-S61 pools whereby ST6Gal1 was further introduced (Fig. 5C). When co-expressing ST6Gal1 and B4GalT1 gene in the MGAT5 overexpressing pools, the peaks for G2F and G2FS1 bi-antennary glycoforms were most prominent. The higher complex tri-antennary N-glycans decorated with two galactoses and terminal sialic acids (FA3G2S1) were also detected although the magnitude was low. Overall, stably co-expressing B4GalT1 and ST6Gal1 genes in the MGAT5 overexpressing pools had no effect on fucose levels while enhanced galactosylation level to 55% and sialylation to 30% (Fig. 5E,F,G). The increment was a lot less than that observed in the G1 + S61 targeted pools (Fig. 3F,G). This could be in part due to the low gene overexpression and clonal variation effect caused by the random integration, highlighting the necessity of using targeted integration technology for cell engineering.

## Discussion

To expand the diversity of N-glycan structures on antibodies, we developed the CHO targeted integration technology, which permits overexpression of glycosyltransferases, to produce antibodies with complex N-glycan structures. By overcoming position effects and thus minimizing clonal variations, our technology enabled the study of gene functions in stably transfected CHO cell pools without the need of isolating clones. Together with the use of multi-cistronic vectors, we were able to engineer both single and multiple genes simultaneously in the glycosylation pathway to produce antibodies with various N-glycan structures. By overexpressing B4GalT1 gene alone, we produced IgGs that were more than 80% bi-galactosylated, which is known to be beneficial for enhancing both ADCC and CDC of the therapeutic antibodies^6,7^. We also demonstrated that combinatorial overexpression of B4GalT1 and ST6Gal1 produced antibodies containing bi-antennary complex N-glycans with more than 70% sialic acids. Highly sialylated glycans are reported to be valuable for increasing antibody clearance time^13^ and anti-inflammatory functions^14,15^. In addition, antibodies with various tri-antennary N-glycans, which have not been reported before, were obtained by overexpressing MGAT5 alone or in combination with B4GalT1 and ST6Gal1. Further investigation of how these novel glycan structures affect antibody functions is in progress. The diverse range of N-glycan structures obtained in this study can be used for optimizing the glycan designs on recombinant antibodies to suit different therapeutic applications.

Cellular demand for sugar precursors and glycosyltransferase levels vary as the N-glycan elongating. Limited substrates or modifying enzymes at any steps of the N-glycosylation pathway will create bottlenecks in N-glycan processing^44,45^. Identifying and mitigating these bottlenecks are crucial for producing higher complex-type N-glycans such as sialylated structures. Via systematic screening of 42 glycosyltransferase genes, we found that overexpression of most genes involved in the nucleotide sugar synthesis and transporter steps had little impacts on the IgG glycan profile, suggesting that early steps of the glycosylation pathway were not limiting the formation of complex-type N-glycans on antibodies. Recent study by Sumit et al. identified that temporal bottlenecks occurred at the galactosylation and sialylation steps in CHO cells by analysing the intermediate metabolites and glycosylated species in antibody production^45^. Our study supported their finding and further demonstrated that co-expression of galactosyltransferase and sialyltransferase was required for obtaining high mAb sialylation. Moreover, our work suggested that the bottlenecks in the glycosylation pathway shifted as the expression of different glycosylation genes was perturbed. The antibodies produced in the wild-type CHO MCL had low galactose and sialic acids. Overexpression of B4GalT1 in the CHO MCL increased galactose but not sialic acid, suggesting galactosylation was the first bottleneck for producing complex glycans in the wild-type CHO cells. In the G1 + S61 targeted pools overexpressing both B4GalT1 and ST6Gal1, further introduction of ST6Gal1 but not B4GalT1 resulted in additional sialylation improvement, suggesting once the galactosylation bottleneck was released, the expression level of downstream sialyltransferase became the limiting factor for further increasing IgG sialylation. Therefore, multiplexed engineering glycosyltransferase genes is required for better control of the N-glycosylation dynamics.

In contrast to some glycoproteins that naturally carry multi-antennary glycoform^46,47^, the number of N-glycan branches on antibodies are often limited to two, partly due to the embedment of the Fc N-glycans within a cavity formed by the two CH2 domains^2,48^. Such restricted configuration also makes it increasingly difficult for enzymes acting on later steps of the glycosylation pathway, such as MGAT4 and MGAT5 to access the growing N-glycans during transition of IgG proteins through the Golgi apparatus. Interestingly, we observed in this study that tri-antennary structure in IgG could be produced by overexpression of MGAT5 or MGAT5B. Consistent with our study, Fukata et al. has previously demonstrated that overexpression of MGAT4 and/or MGAT5 genes could increase multi-antennary sugar chains in human interferon (IFN)-γ^49^. We speculate that the access to glycosylation sites on proteins in the ER is related to the abundance of enzymes in the Golgi. As Golgi-residing enzymes can continuously recycle back to the ER^50^, enriching MGAT5 level in Golgi may also potentially increase its distribution in ER, thus allowing them to readily access the glycosylation sites on the folding proteins.

We observed a low incorporation level of galactose into the tri-antennary but not bi-antennary N-glycans when stacking expression of B4GalT1 in the MGAT5-overexpressing cells. This suggested that galactosylation might be directed more favourably to the bi-antennary structures than the tri-antennary ones. In addition, the proportion of tri-antennary structures are reduced with increased B4GalT1 protein levels. Hence, we suspect a potential competition between tri-antennary formation and galactosylation. The presence of galactose residues on the bi-antennary branches may inhibit the addition of the third sugar chain catalysed by MGAT5. A computational simulation of N-glycosylation pathway predicted galactosyltransferase activity could control the final level of antennarity of glycoproteins^51^. The study later demonstrated the enrichment of tri- and tetra-antennary glycans of human chorionic gonadotropin only in the absence of B4GalT4 isoenzyme. The relationship between galactosyltranferases and branching enzymes remains to be investigated. Such understanding will enable better designs of glyco-engineering strategies in the future.

Our work gained insights into the contribution of individual isoenzymes in forming complex N-glycans on antibodies. Single knockout of the B4GalT1 gene resulted in only partially reduction of galactosylation in EPO and Fc-fusion EPO^27^, suggesting a potential involvement of other family members. In our study, we identified that overexpression of B4GalT2 gene in CHO cells could moderately produce more galactosylated antibodies. This provides a new opportunity to utilize B4GalT2 gene for N-glycan-engineering purposes in the future as well as further investigate the involvement of other isoenzymes. In addition, increasing evidence has suggested that the isoenzymes regulate N-glycan formation in a protein specific manner. For example, a study by Qi et al. demonstrated that sialylation of EGFR was highly affected by ST3Gal6 level, but not other isoenzymes^52^. On the other hand, single knockout of three α-2,3 sialyltransferase isoenzymes in CHO cells revealed both ST3Gal4 and ST3Gal6 were important for EPO protein sialylation^29^. In contrast, our study indicated that ST3Gal4 played more important roles than ST3Gal3 and ST3Gal6 in IgG sialylation. We also observed that both α-2,6 sialyltransferase enzymes were more efficient in incorporating sialic acid into the growing glycans than α-2,3 sialyltransferases, although they are not expressed in CHO cells naturally. This finding suggested that α-2,6 sialyltransferase family members may exhibit higher specificity toward antibodies.

## Methods

### Cell culture and media for maintenance of CHO K1 master cell line (MCL)

The CHO K1 master cell line (MCL) was generated by nucleofection of a landing pad vector into CHO K1 cells (ATCC), followed by screening clones for single copy integration by southern blotting. The landing pad vector expressed a hygromycin resistant gene (HYG) using a chimeric promoter (ChiP) which consisted of the murine CMV enhancer (M11788), the hCMV core promoter and the hCMV intron A (M60321). The HYG expression cassette was flanked by FRT3 and FRT. An impaired puromycin resistant gene lacking start codon ((ATG-)Puro) followed by the simian virus 40 (SV40) polyadenylation signal (pA) was placed downstream of FRT for selecting correct cassette exchange by RMCE (Fig. 1A). It was confirmed that the MCL contained only one copy of landing pad vector at a single integration site by southern blotting and targeted locus amplification (TLA) analyses (Cergentis). The MCL was grown in a protein-free medium (maintenance media) consisting of 50% HyQ PF (GE Healthcare Life Sciences) and 50% CD CHO (Thermo Fisher Scientific) supplemented with 1 g/L sodium carbonate (Sigma), 6 mM glutamine (Sigma) and 0.1% Pluronic F-68 (Thermo Fisher Scientific) in a humidified Kuhner shaker (Adolf Kühner AG) with 8% CO_2_ at 37 °C. Routine subculture was conducted every 3 to 4 days by seeding cells at density of 3 × 10^5^ cells/mL in 15 mL of fresh medium in 125 mL shake flasks (Corning). Cell density and viability were determined by trypan blue exclusion method on Vi-Cell XR viability analysers (Beckman Coulter).

### Constructing targeting plasmid vectors for expression of mAb and human glycosyltransferase genes

The control targeting vector carrying an expression cassette of FRT3-LC-IRES-HC-DsRed-IRES-FRT was constructed by Genscript. LC and HC represented the rituximab light chain cDNA and heavy chain cDNA, respectively. IRES represented the wild-type encephalomyocarditis virus (EMCV) internal ribosome entry site. Three unique restriction sites, MfeI, XhoI and PmeI were included between HC and IRES-DsRed for insertion of more expression units. To construct targeting vectors expressing various single or double human glycosyltransferase genes, two basic vectors were first constructed by inserting one synthesized unit IRES-GE1 using MfeI and XhoI followed by inserting the second synthesized unit IRES-GE2 using XhoI and PmeI. GE1 and GE2 represented specific human glycosyltransferase genes. The IRES element used upstream of GE1 and GE2 was either the wild-type EMCV IRES or a mutated IRESv18 variant with reduced strength for expressing a gene. Two restriction sites, ClaI and NruI were created by mutating the six bases in front of the 10th ATG in the EMCV IRES upstream of GE1 and GE2, respectively during gene synthesis. Targeting vectors expressing other single or double human glycosyltransferase genes were subsequently constructed by inserting the synthesized human glycosylation genes between ClaI and XhoI or NruI and PmeI. Synthesis of IRES-GE1, IRES-GE2, other glycosyltransferase genes and inserting them into the control vector or the two basic vectors for expressing single or double glycosylation genes were all done by Genscript. The sequences of the wild-type IRES, IRESv18, FRT and FRT3 were described in previous studies^53,54^. The sequences of DsRed gene and pA were cloned from the pIRES2-DsRed vector (Clonetech) and the pcDNA3.1 (+) vector (Thermo Fisher Scientific), respectively. The rituximab LC cDNA and HC cDNA were designed based on the amino acid sequences published in the international ImMunoGeneTics information system (IMGT). The sequences of human glycosyltransferase genes were retrieved from NCBI (Table 1). The vector expressing enhanced FLP recombinase (FLPe) was synthesized by Genscript (Fig. 1A). The sequence of murine CMV enhancer and promoter (mCMV) (M11788) was described in a previous study^55^. The sequence of FLPe gene was from the pCAGGS-FLPe vector (Gene Bridges). The sequence of SpA was from the pcDNA3.1 (+) vector (ThermoFisher). The three plasmid vectors for overexpressing B4GALT1, ST6GAL1 and the combination of B4GALT1 and ST6GAL1 in stable transfections were constructed using a previously described tricistronic vector^56^. This tricistronic vector expressed LC, HC and Blasticidin (Bla) resistant gene in one transcript through the use of two wild-type EMCV IRES. The LC gene is under the control of the chimeric promoter (ChiP). Expression of HC and Blasticidin (Bla) resistant gene were driven by the wild-type EMCV IRES. The two vectors expressing B4GALT1 or ST6GAL1 were constructed by replacing the region LC-IRES-HC in the tricistronic vector with B4GALT1 or ST6GAL1. The vector expressing the combination of B4GALT1 and ST6GAL1 was constructed by replacing the LC with B4GALT1 and the HC with ST6GAL1, respectively. All restriction enzymes used were purchased from New England Biolabs. Plasmids were all propagated using chemically competent DH5α *E.Coli* cells (Thermo Fisher Scientific).

### Generating stable mAb-producing cell lines via recombinase-mediated-cassette-exchange (RMCE) and random integration

The MCL was co-transfected with an appropriate targeting vector and a vector expressing FLPe using Amaxa SG Cell Line 4D-Nucleofector X Kit and program FF-137 (Lonza). In each transfection, 1 × 10^7^ cells were transfected with 5 µg of targeting plasmid vector and 5 µg of FLPe plasmid vector in circular format. The transfected cells were then re-suspended in 2 mL of maintenance media preloaded in 6-well suspension culture plates (NUNC) and incubated in the static IncuSafe incubators (Sanyo). At 24 h post-transfection, they were collected by centrifugation (100 × g, 5 min) and re-suspended in 15 mL of protein-free maintenance medium in 125 mL shake flasks in the humidified Kuhner shaker (Adolf Kühner AG) with 8% CO_2_ at 37 °C. Four days later, the transfected cells were subjected to selection in the maintenance media containing Puromycin (InvivoGen) at 20 µg/mL. Selection was continued for two weeks by passaging in the selection medium every 3 to 4 days. Stably transfected cell pools were deemed established when cell viabilities recovered over 95%.

The protocol for further transfection of the three multi-cistronic vectors expressing B4GALT1, ST6GAL1 and the combination of these two genes respectively into the targeted pools already expressing either the combination of B4GALT1 and ST6GAL1 or MGAT5 was the same as that described for RMCE with slight modifications. In each transfection 5 µg of linearized plasmids were transfected to 1 × 10^7^ cells. After incubating in 2 mL of protein-free medium in the 6-well suspension culture plates (NUNC) overnight, the transfected cells were collected by centrifuge at 100 × g for 5 min and then resuspended in 15 mL of protein-free medium supplemented with blasticidin (Thermo Fisher Scientific) at 20 µg/mL. Passaging in selection medium was subsequently carried out every 3 to 4 days until cell viabilities recovered over 95%.

### Characterization of growth and productivity of stable pools

Stable cell pools were subjected to seven-day fed-batch production by seeding 30 mL of cultures at viable cell density of 3 × 10^5^ cells/mL in 50 mL tube spin (TPP) in the humidified Kuhner shaker (Adolf Kühner AG) with 8% CO_2_ at 37 °C. 3 mL of Ex-Cell Advanced CHO Feed 1 (with glucose) (Sigma) and 400 µL 45% (w/v) D-glucose (Sigma) were added at day 5. Cell density, viability and antibody titer were monitored at day 3, 5 and 7 using the Vi-Cell XR viability analyzer (Beckman Coulter) and an IMMAGE 800 immunochemistry system (Beckman Coulter), respectively. The IMMAGE 800 immunochemistry system utilized anti-human Fc region antibodies for IgG quantification. The specific mAb productivity (qP) in the exponential phase of cultures was calculated as the difference in mAb concentration between day 5 and 7 divided by the integrated viable cell density (IVCD) which was determined based on the trapezoidal method. Two sets of ten million cells were collected from each culture at day 5 for analysis of mRNA and protein levels, respectively. Flow cytometry was performed at day 5 on BD FACSCalibur to determine the homogenous expression of the DsRed protein in the stable pools. Flow cytometry data were analyzed using FlowJo software. Culture supernatant was harvested at day 7, by centrifuging at 5000 × g over 10 min to remove cells and use for N-glycan analysis.

### Analysis of genomic DNA, mRNA and protein levels

To confirm correct cassette recombination, crude genomic DNA (gDNA) was extracted from cell pellets using PureLink Genomic DNA Mini Kit (Thermo Fisher Scientific) according to the manufacturer’s protocols. 100 ng of gDNA was used as template for PCR using 2X REDiant Master Mix (1st Base) with PCR condition according to the manufacturer’s protocols. PCR primers for 5′ and 3′ junction PCRs are listed in the Supplementary Table S1. PCR products were visualized on 1% agarose gels stained with ethidium bromide.

Total RNA was isolated using RNeasy Mini Kit (Qiagen) from ten million cells collected from each fed-batch culture. Analysis of the mRNA levels for human glycosyltransferase genes (Table 1) and β-actin was done using either reverse transcription polymerase chain reaction (RT-PCR) or quantitative real-time PCR (qRT-PCR) as described previously^53^. Primer sequences used for mRNA analysis were listed in Supporting Table S2. For RT-PCR, 1 µg of total RNA from each sample was used as template for cDNA synthesis. The PCR products corresponding to each human glycosyltransferase gene were further analyzed by electrophoresis on 2% agarose gel and visualized by ethidium bromide staining.

To carry out Western blotting for protein level analysis, ten million cells were first homogenized in the CelLytic M (Sigma) supplemented with Halt Protease and Phosphatase Inhibitor Cocktail (Thermo Fisher Scientific). 10 µg of each protein sample quantified by Pierce BCA protein assay (Thermo Fisher Scientific) was fractionated on 4–12% gradient PAGE gel (Thermo Fisher Scientific), followed by transfer to a PVDF membrane using iBlot (Thermo Fisher Scientific). The membrane was then blocked for 1 h with a blocking buffer comprising of 1 × Tris buffered saline with 0.1% Tween20 (1st Base) with 5% non-fat milk. The membrane was probed with primary mouse anti-ST6Gal1 antibody (1:500) (R&D Systems), or GAPDH (1:1000) (Abcam) overnight at 4 °C. After washing with TBST, the membrane was incubated with 1:5000 corresponding diluted secondary antibody (Promega) in blocking buffer for 1 h at room temperature. After washing, the membrane was visualized using Amersham ECL Western blotting detection reagents and analysis system (GE Healthcare Life Sciences) according to the manufacturer’s instructions.

### Antibody purification and N- glycosylation analysis

mAb in the culture supernatant was purified using MabSelect SuRe Protein A column (GE Healthcare Life Sciences) on a GE AKTA explore 100 (GE Healthcare Life Sciences). The purified mAbs were analyzed for the N-glycosylation using hydrophilic interaction chromatography (HILIC) with fluorescence detection. The protocols for protein A purification and N-glycosylation analysis have been described in a previous study^53^. The data obtained from N-glycosylation analysis was analyzed with the UNIFI Biopharmaceutical software platform (version 1.8). The N-glycan structures were assigned to peaks based on the alignment of observed and expected glucose unit (GU) values. The peak assignment followed the method described in a previous study^53^. The abundance of each structure was expressed as percentage of total peak area. The level of galactosylation, fucosylation were calculated as described in a previous study^57^. Galactosylation is the percentage of the number of galactose residues in G1, G1F, G2 and G2F in the total number of galactose residues in G0, G0F, G1F, G1, G2 and G2F if they are fully galactosylated. Fucosylation is the percentage of fucosylated species (G0F, G1F, G2F) in the total sum of fucosylated and afucosylated species (G0, G1, G2). Sialylation was the sum of the relative abundance of mono- and bi-sialylated species. The high mannose was the relative abundance of M5 structure on mAb.

## Supplementary Information

Supplementary Information.
