# Supplementary material for: Multiplexed engineering glycosyltransferase genes in CHO cells via targeted integration for producing antibodies with diverse complex-type N-glycans
Source: Sci Rep. 2021 Jun 21;11:12969. doi: 10.1038/s41598-021-92320-x (PMC8217518; doi:10.1038/s41598-021-92320-x)
Supplement: Supplementary file 1 — Supplementary Information. [file 41598_2021_92320_MOESM1_ESM.pdf]

Supplementary information

Title

Multiplexed engineering glycosyltransferase genes in CHO cells via targeted integration for producing antibodies with diverse complex-type N-glycans

Authors and affiliations

Ngan T.B. Nguyen, Jianer Lin, Shi Jie Tay, Mariati, Jessna Yeo, Terry Nguyen-Khuong, Yuansheng Yang*

Bioprocessing Technology Institute, Agency for Science, Technology and Research (A*STAR), Singapore, Singapore

*Corresponding author: Dr. Yuansheng Yang, yang_yuansheng@bti.a-star.edu.sg.

Supplementary table S1. List of primers for junction PCR analysis.

	Name	Sequence (5′-3′)
1	**P1**	AGGCAGCTGAGTTGTTGTATTCTGATAAGA
2	**R1**	ACTGAAGCGAACAGGGACTCCAGAAGCCAG
3	**P2**	CAACGAGGACTACACCATCGTGGAGCAGTA
4	**R2**	CGACGCGCGTGAGGAAGAGTTCTTGCA

Supplementary table 2. List of primers for RT-PCR analysis.

	Gene	Forward primer (5′-3′)	Reverse primer (5′-3′)
1	GALE	GGCACGGGCACAGGCTATTCA	CTGGGGTTGGCGTAACAGGCT
2	HGNE	TCCCTCCCTTGTGATCCTCTCCG	GGCGGGGTCAACCAAATCCGA
3	NANS	TGCAGTGTACCAGCGCATACCC	GTTCCAACACCTTGGCCCCCA
4	NANP	GGGGACTGTGTGATGGTCGGTG	GGGGACTGTGTGATGGTCGGTG
5	CMAS	CCGCACCTGGCAGCCCTAATTC	CCCATACACTCTGGAAGGCCCC
6	CST	GCTGCCCCGAGAGACAATG	CGGCTGTGGTTGAAAAGTAGAGT
7	UGT	GGTGCAGCCAAAGCCATAGCC	GGCAGAAAGGGCTCCGTGATGA
8	UGNT	GAACCCCTTCACCCTGTGGCA	CTTCTTGCTGTCCTTCTGAGGCT
9	GFT	TGCGGTATCATCATCGGGGGC	TGGCGTTGAGCGAGACACAGA
10	GANC	TTGCGGAGCTGACATAGGCGG	GGGCTCTCGTCGCTTGGTGTT
11	MANEA	TCCACAAGGGAGACACAACCCT	ACCAAGAGAGGGCTAGTACACCA
12	MAN1A1	TGACAAGGCGCCGTTCAGAGG	TGCCCTTTTCTCGCGGATGGC
13	MAN1B	ACCAGTCCCTATTCCCAACCT	TGAGTTCATTATGTCCCCACCCA
14	MAN1C1	TTTCTCCGGGGGCATGATCGC	AGCGGGCGTATGACTCGTGAC
15	MAN2A2	TCCCCCTCCAGGCCAACTTCT	CATCAGCCGCCGGTCCAAGAT
16	MAN2B1	GTTCCCAGGTGGTTCGCCTGT	AGCGTCCCTTTGTCTCCAGCG
17	MAN2B2	CATGTACGCAACGCACCTGGC	GCGGGTTGTAGACCGAGGCAA
18	MAN2C1	TGGACTGCTGGCTGCACTTGT	TGAGCTTCAGGCGGTTGTCCC
19	MGAT1	TGGAATGCCCTGCTGCTCCTC	AGGCGAATCACTTCCCGGGTG
20	MGAT2	CAGCTGATCGCCGGGGTGAAT	AAGCGGCATTCTTCGGCAGGT
21	MGAT3	TCCACATGCGCAAGTCGCTCT	TCGAGCCCATACACTGCCTGC
22	MGAT4A	TGGGCATTCCCACAGTGAAGAGA	CTCCAGGTTGGCTACAACACCA
23	MGAT4B	CCTTCCTGACGCTGCTGCTCT	TCCCGCTGGTAAACGTCCACA
24	MGAT4C	GGGGAAACCACCTTCAACAGGAG	CGTTTTCCCCAACATCTAGGGCT
25	MGAT5	ATTTACGTGCGAGGGGATGCT	ATTTACGTGCGAGGGGATGCT
26	MGAT5B	GAACCACGGCCTCTTACCGCA	AACCATTGGCGATGGCCTCCA
27	B4GALT1	ATTGGGGCTGGGGAGGAGAAGA	TCATGCGACACCTCCCGACCA
28	B4GALT2	GTGGCCTTGTTTGCCAGCAGC	ATGACGGCCACGAGGAAGTGC
29	B4GALT3	GCCGAGATCAGGGACCGACAT	GCAGACCTTGAGGAGCTGGAGG
30	B4GALT4	AGAGGGTCGCCATCCTCGTTC	TGCCATAATCCAGCTGCTGCC
31	B4GALT5	TCTCTCTCGTCCTCGCTGCTGT	CGTTGTCCCGGATCAGAATGCCT
32	B4GALT6	ACGGATGTGGAGAAATGCCACG	TGTCAGCCCACTTACACCACCA
33	B4GALT7	CGGCATCCTGCTGCTCTCCAA	ATGCGCCGGTAGAACTCGTCG
34	ST3GAL1	AAGTGCTCACCTTCCTCGTGC	GGACCATCTGCTTGGGGAACC
35	ST3GAL2	CAACCGTGGGCTTTGAGCAGG	GAATCGGATCTGCCCCGTGGAC
36	ST3GAL3	CCCCAGGAGAAGCCTGTTGCAG	TTCAGGAGGAAGCCCAACCGA
37	ST3GAL4	TGGCTCTGGTCCTGGTCGTCA	CTGGAGGCACGGCTCCTTCTT
38	ST3GAL5	TCAGTTTGGCGGGTTTTGGATA	TCACCACTCCCTCTTTGACCAG
39	ST3GAL6	TGGGGAACGAATGTCTATTGGG	ATCAGCCGCACACAGAAAAGG
40	ST6GAL1	AAGCTGCACCCCAATCAGCCC	TCCACCTGGTCACACAGCGTC
41	ST6GAL2	AGGCGGGTGAAGAAGAGGCAC	AGGCGCGGGTTCAGCATTTTG
42	FUT8	TGCTCACCAACCCCGAACTGC	GGCCCGTCCTTCCCAATTTCCT
43	ACTIN	AGCTGAGAGGGAAATTGTGCG	GCAACGGAACCGCTCATT

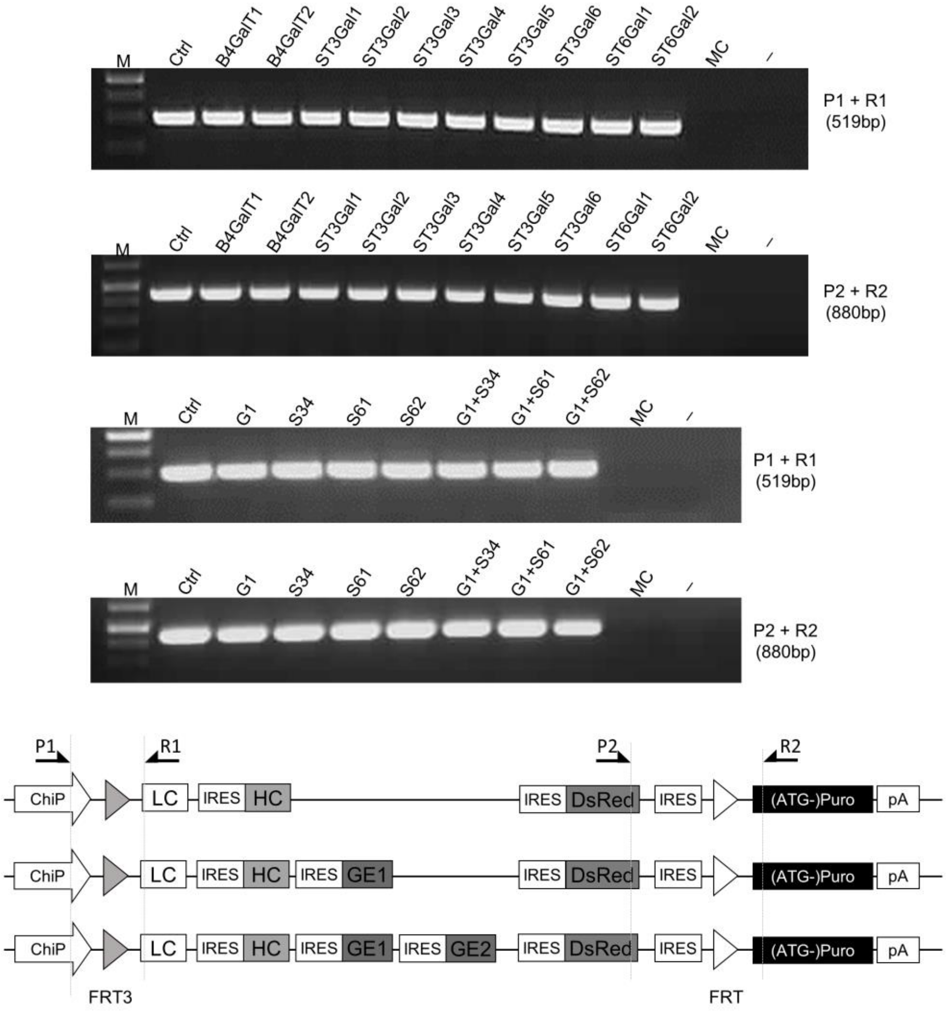

Supplementary Figure S1. Junction PCR performed on gDNA of stably transfected pools to show correct cassette exchange for stable pools derived from RMCE. PCR product generated using primer pair P1 and R1 confirmed integration across the FRT3 junction; and PCR produce generated using P2 and R2 set confirmed integration across the FRT junction. Uncropped gel images can be found in Supplementary figure S6.

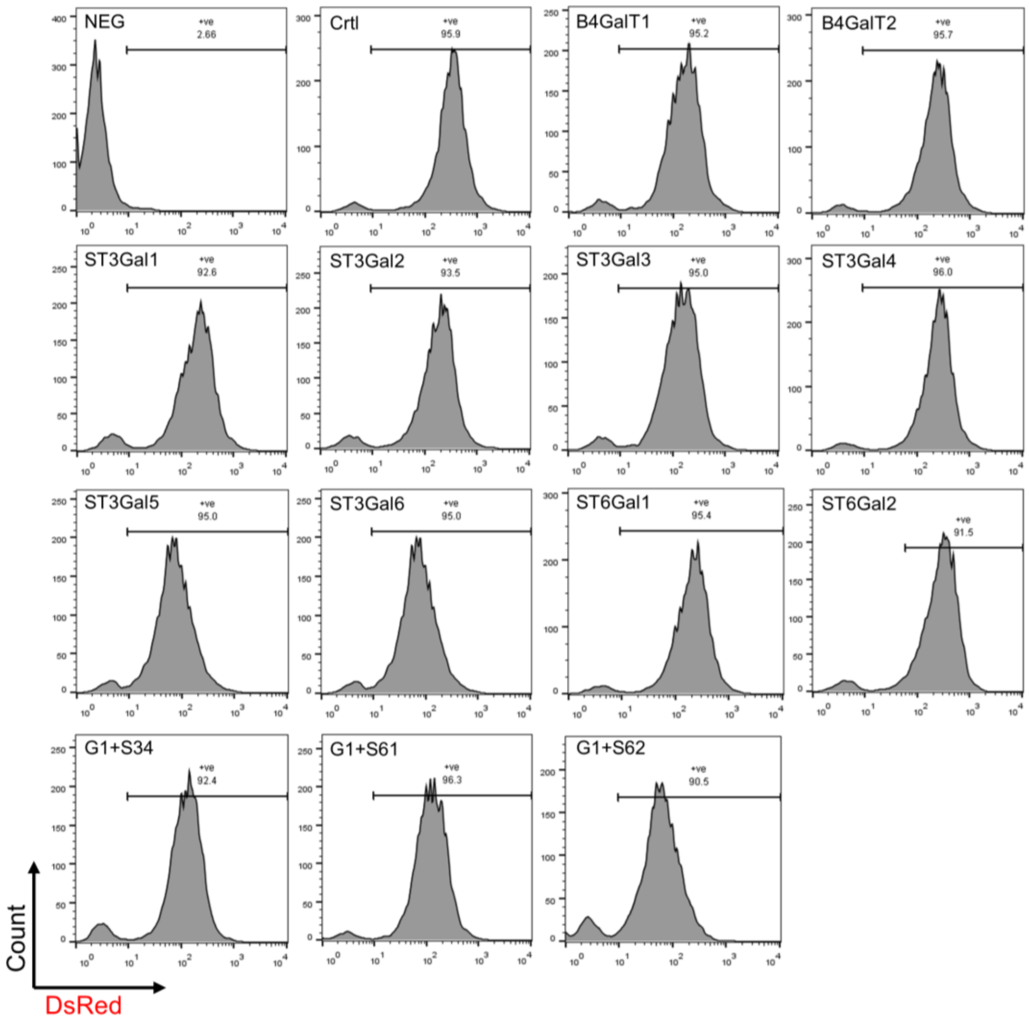

Supplementary Figure S2. FACS analysis of the stably transfected pools derived from RMCE at day 5 during fed-batch production. Plots of DsRed fluorescence vs count. Most pools displayed homogenous expression of DsRed fluorescene protein with more than 90% of cells were DsRed positive.

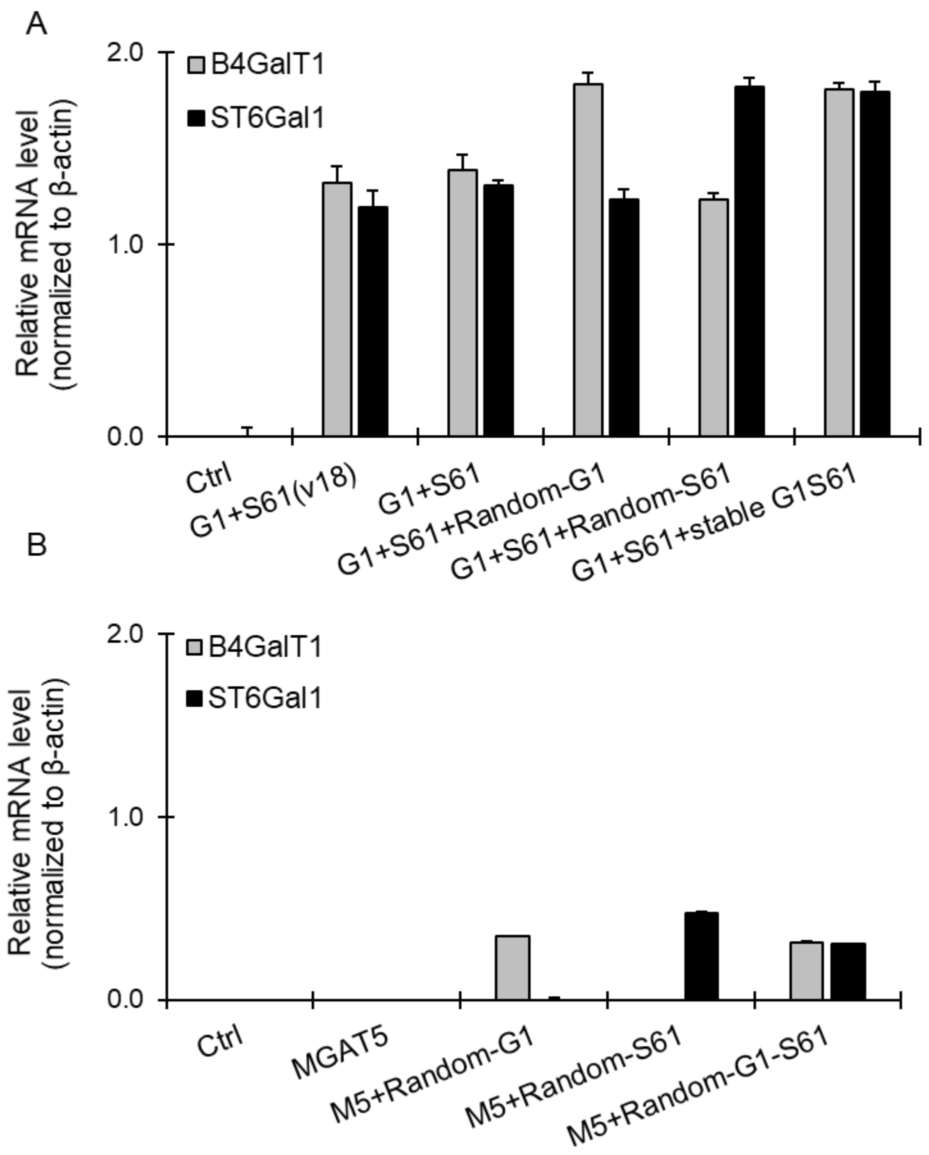

Supplementary Figure S3. qRT-PCR analysis for B4GalT1, ST6GalT1 transcript levels upon random integration of B4GalT1, ST6Gal1 gene individually or in combination into (A) G1+S61 targeted pool and (B) MGAT5 targeted pool. β-actin (ACT) was used as an internal control.

(a)

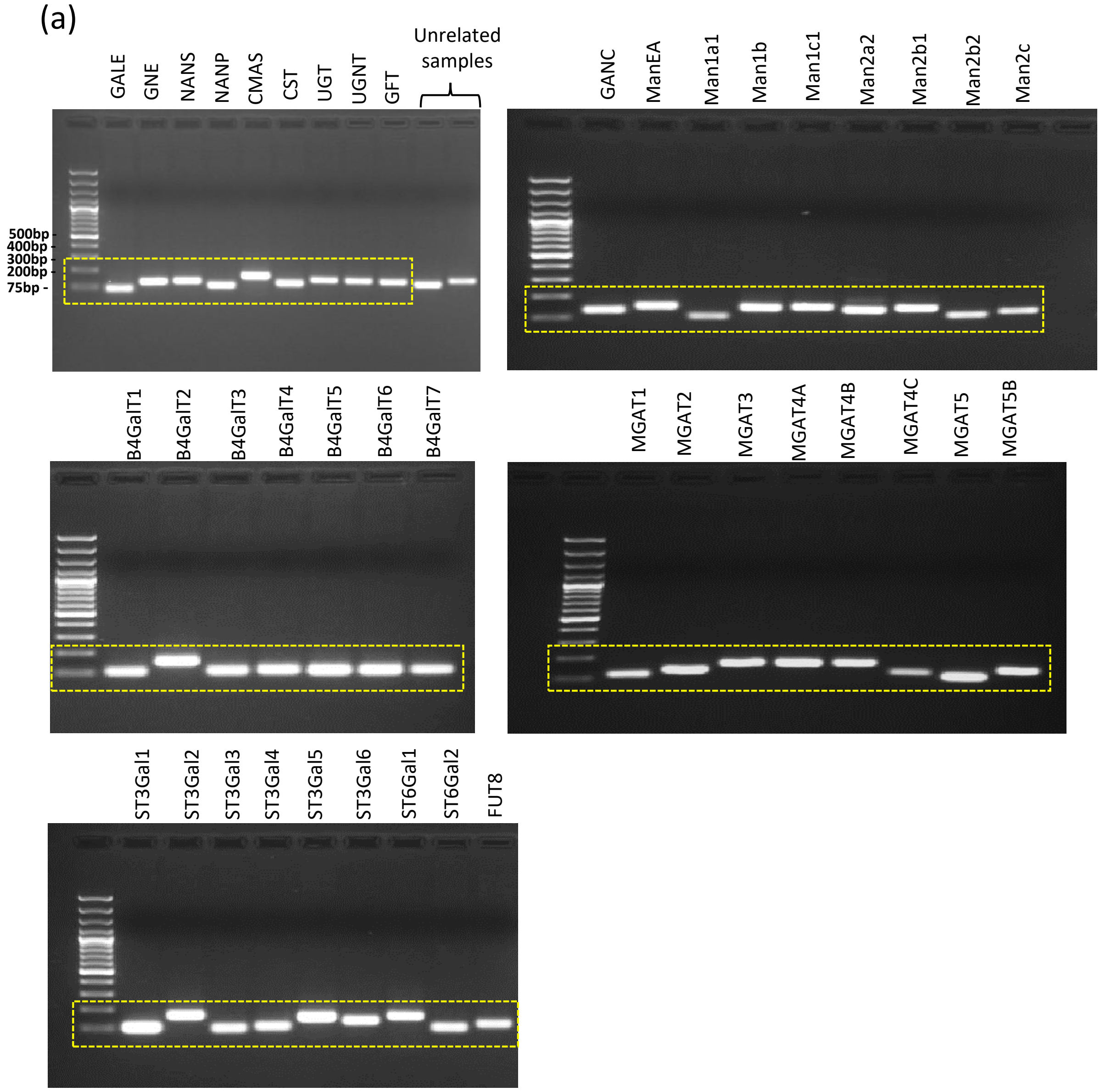

Unrelated samples
GALE
GNE
NANS
NANP
CMAS
CST
UGT
UGNT
GFT
500bp
400bp
300bp
200bp
75bp
GANC
ManEA
Man1a1
Man1b
Man1c1
Man2a2
Man2b1
Man2b2
Man2c
B4GalT1
B4GalT2
B4GalT3
B4GalT4
B4GalT5
B4GalT6
B4GalT7
MGAT1
MGAT2
MGAT3
MGAT4A
MGAT4B
MGAT4C
MGAT5
MGAT5B
ST3Gal1
ST3Gal2
ST3Gal3
ST3Gal4
ST3Gal5
ST3Gal6
ST6Gal1
ST6Gal2
FUT8

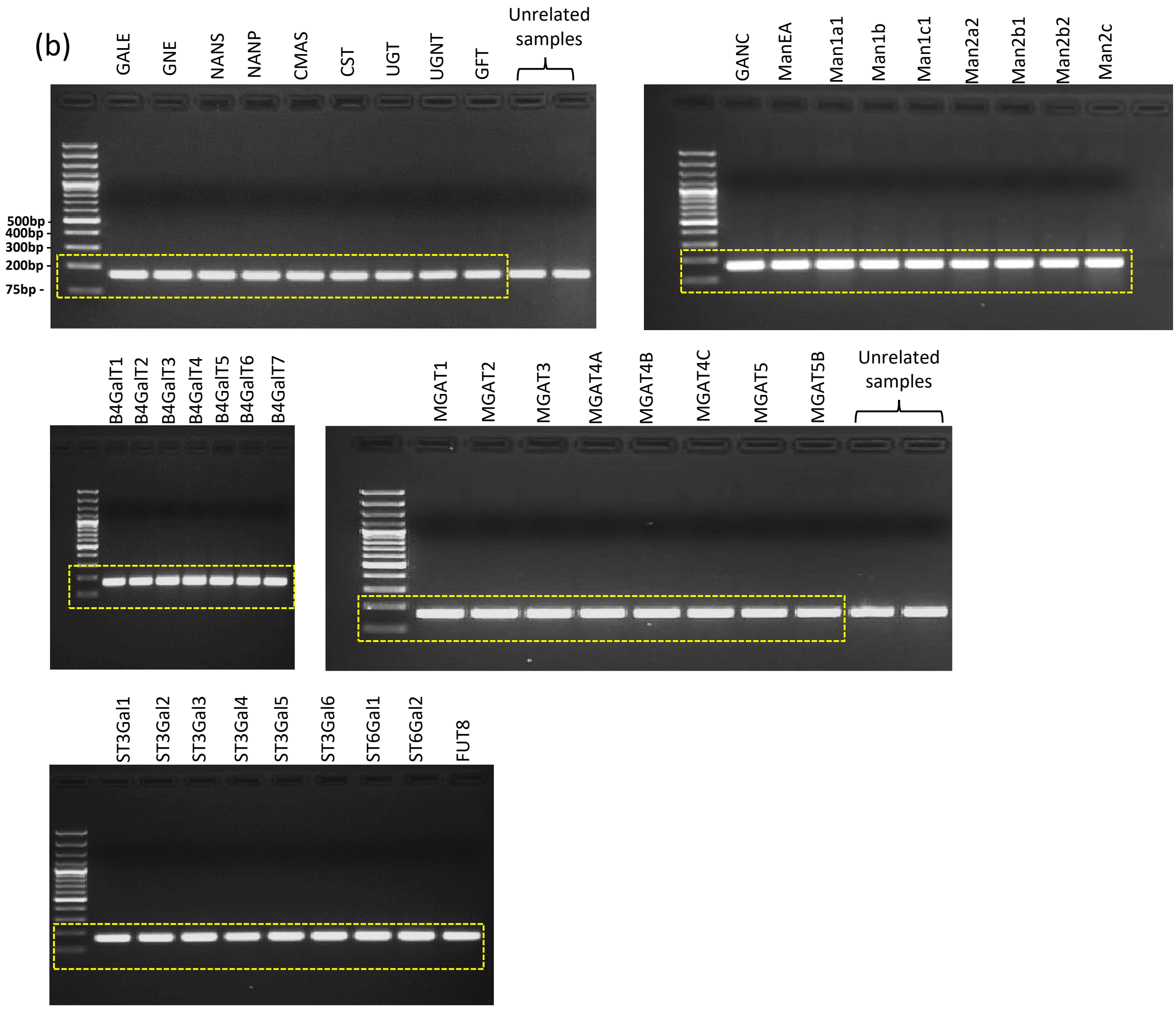

Supplementary Figure S4. Uncropped images of DNA gels shown in figure 2A. The yellow dotted lines indicated the corresponding regions in the main figure 2A. (a) The mRNA of each glycosyltransferase gene from the corresponding stable pools was analysed via RT-PCR and resolved in 2% agarose gels. (b) The mRNA of internal control (β-actin) of the corresponding stable pools was also analysed via RT-PCR and resolved in 2% agarose gels. No changes were done to the images' brightness and contrast.

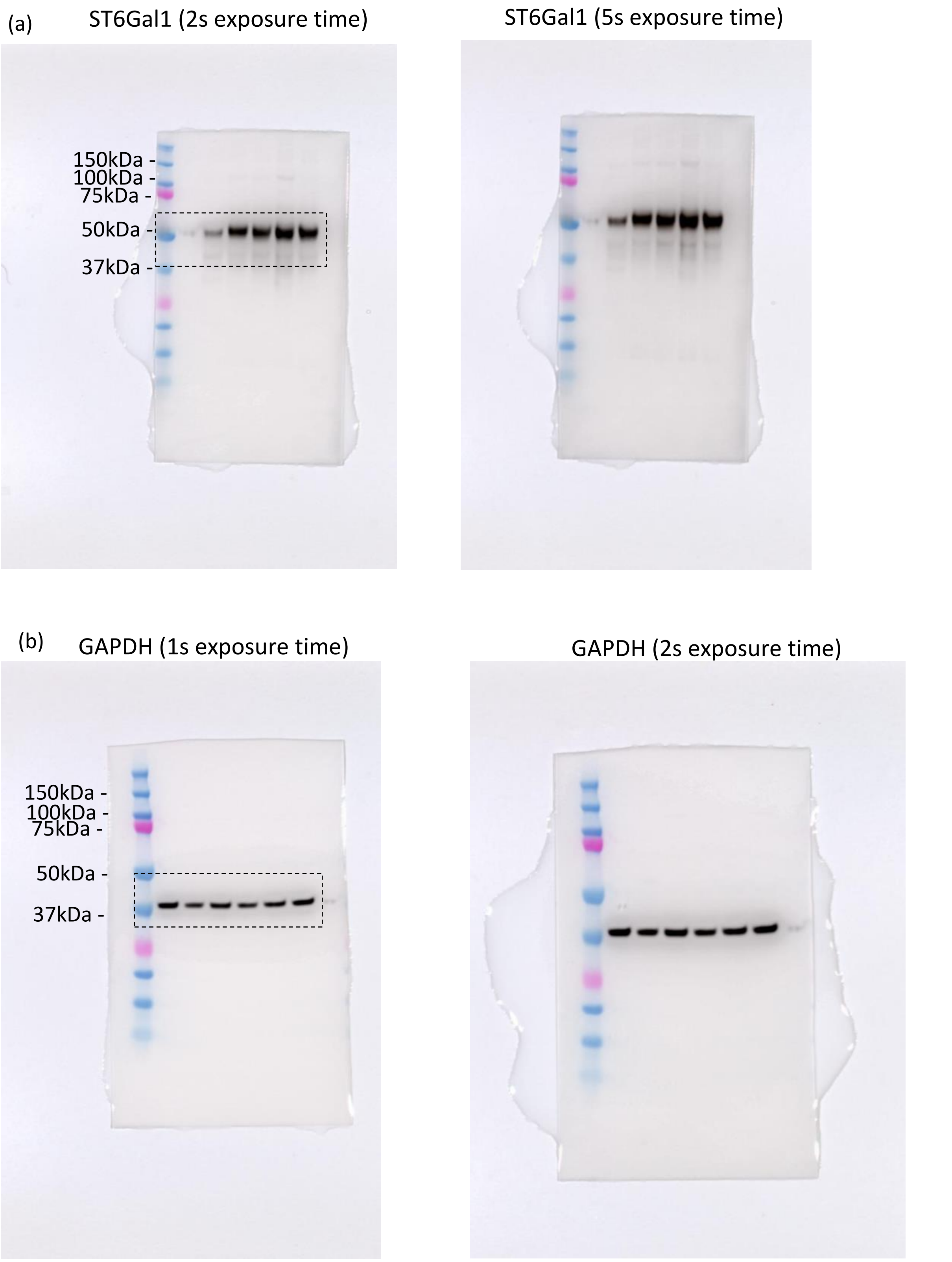

Supplementary Figure S5. Samples are separated on two SDS-PAGE gels. Each gel was then immunoblotted with the respective antibodies. (a) Uncropped image for figure 4A (upper panel). (b) Uncropped image for figure 4A (lower panel). The dotted lines indicated where the images were cropped from the original blots and presented in the main figure 4A. The images'

brightness and contrast were not adjusted, though the images were recoloured into grayscale. Two different exposure time for each blot were included.

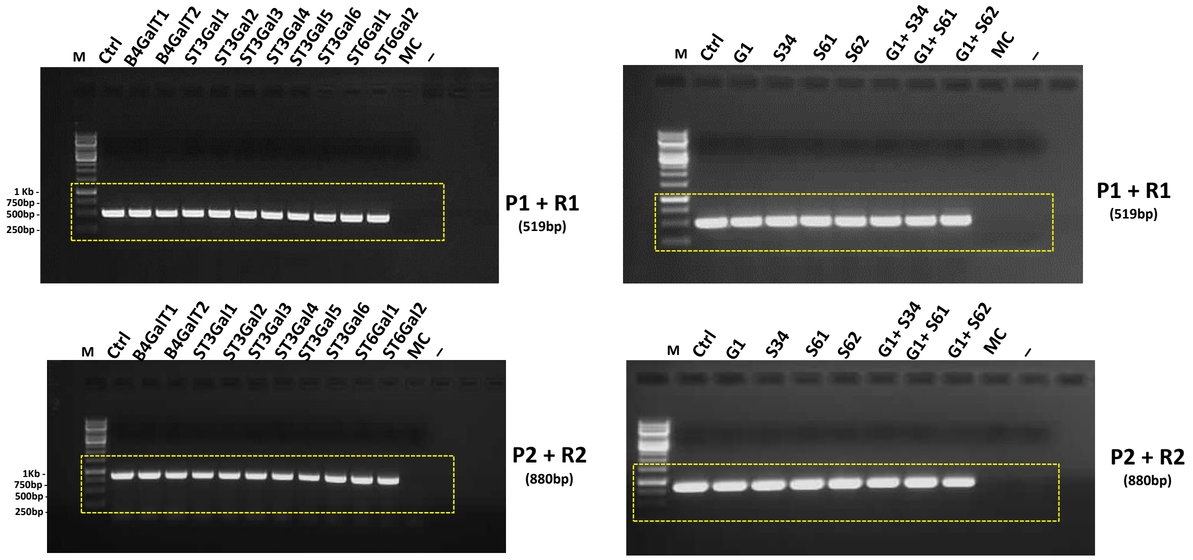

Supplementary Figure S6. Uncropped images of DNA gels shown in supplementary figure S1. The yellow dotted lines indicated the corresponding regions in the figure S1. No changes were done to the image's brightness or contrast.
